# A Circulating microRNA Signature Predicts Age-Based Development of Lymphoma

**DOI:** 10.1371/journal.pone.0170521

**Published:** 2017-01-20

**Authors:** Afshin Beheshti, Charles Vanderburg, J. Tyson McDonald, Charusheila Ramkumar, Tatenda Kadungure, Hong Zhang, Ronald B. Gartenhaus, Andrew M. Evens

**Affiliations:** 1 Division of Hematology/Oncology, Molecular Oncology Research Institute, Tufts Medical Center, Boston, Massachusetts, United States of America; 2 Harvard NeuroDiscovery Center, Massachusetts General Hospital, Boston, Massachusetts, United States of America; 3 Cancer Research Center, Hampton University, Hampton, Virginia, United States of America; 4 Department of Cell Biology and Development, University of Massachusetts Medical School, Worcester, Massachusetts, United States of America; 5 Marlene & Stewart Greenebaum Cancer Center, Department of Medicine, University of Maryland, Baltimore, Maryland, United States of America; Cleveland Clinic, UNITED STATES

## Abstract

Extensive epidemiological data have demonstrated an exponential rise in the incidence of non-Hodgkin lymphoma (NHL) that is associated with increasing age. The molecular etiology of this remains largely unknown, which impacts the effectiveness of treatment for patients. We proposed that age-dependent circulating microRNA (miRNA) signatures in the host influence diffuse large B cell lymphoma (DLBCL) development. Our objective was to examine tumor development in an age-based DLBCL system using an inventive systems biology approach. We harnessed a novel murine model of spontaneous DLBCL initiation (Smurf2-deficient) at two age groups: 3 and 15 months old. All Smurf2-deficient mice develop visible DLBCL tumor starting at 15 months of age. Total miRNA was isolated from serum, bone marrow and spleen and were collected for all age groups for Smurf2-deficient mice and age-matched wild-type C57BL/6 mice. Using systems biology techniques, we identified a list of 10 circulating miRNAs being regulated in both the spleen and bone marrow that were present in DLBCL forming mice starting at 3 months of age that were not present in the control mice. Furthermore, this miRNA signature was found to occur circulating in the blood and it strongly impacted *JUN* and *MYC* oncogenic signaling. In addition, quantification of the miRNA signature was performed via Droplet Digital PCR technology. It was discovered that a key miRNA signature circulates throughout a host prior to the formation of a tumor starting at 3 months old, which becomes further modulated by age and yielded calculation of a ‘carcinogenic risk score’. This novel age-based circulating miRNA signature may potentially be leveraged as a DLBCL risk profile at a young age to predict future lymphoma development or disease progression as well as for potential innovative miRNA-based targeted therapeutic strategies in lymphoma.

## Introduction

Diffuse large B-cell lymphoma (DLBCL) is the most common form of non-Hodgkin lymphoma (NHL), accounting for approximately one-third of patients diagnosed in the United States [1, 2]. Although DLBCL is curable in the majority of patients, approximately 35–40% of patients die due to disease progression, while acute and late toxicities remain an issue among treated patients [1, 2]. Detection and treatment options for DLCBL are typically developed by observational clinical studies rather than measurable biological differences [3, 4]. This has resulted in a general lack of precision medicine approaches to date in current DLBCL therapeutic paradigms [3, 5]. Varied molecular factors, however, are emerging as potential prognostic and therapeutic targets in DLBCL [2, 6]. A specific transcription factor, *JUN*, was shown to be frequently activated in DLBCL and highly upregulated in a large number of genes and significantly contributed to DLBCL growth through interaction with the microenvironment [7]. Additionally, we recently showed in an analysis utilizing The Cancer Genome Atlas (TCGA) that *JUN* impacted older DLBCL patients [8]. In general, continued knowledge is needed to identify specific molecular changes and potential actionable pathways for prognosis and therapeutic targets in DLBCL.

MicroRNAs (miRNAs) are small non-coding RNAs that impact post-transcriptional gene expression and are increasingly being recognized in cancer, including NHL, as important in pathogenesis, prognosis, and therapy [9–14]. Each miRNA can target hundreds of mRNAs, which predicts that over half of the existing human transcriptome is regulated by miRNAs [15, 16]. Not only do miRNAs impact the transcriptome, they are now known to target and regulate proteins and DNA [17, 18]. Recent studies have started to implicate miRNAs in driving DLBCL progression [19–21], but which specific miRNA signatures impact DLBCL development or progression remains to be fully delineated. MicroRNAs have also been implicated with age. Evidence suggests a tissue-specific coordinated pool of miRNAs contribute to the “hallmarks of aging” [22]. Overlap exists between the miRNA signatures in DLBCL and age related miRNAs, but little is reported on how all these factors uniformly effect DLBCL development, progression, and survival of patients. Further, the impact of where and how the miRNAs affecting these factors is not understood. Recent evidence showed distinct miRNA signatures in the blood that arises from tumor burden [10, 23, 24]. These circulating miRNAs are highly stable, resistant to degradation, and have potential to be used as a non-invasive novel therapeutic strategy [23, 25]. Additionally, there have also been distinct circulating miRNAs associated with age-related changes that can impact a variety of diseases included DLBCL [26–28]. Single circulating miRNAs are an attractive minimally invasive tool for potential use as biomarkers for lymphoma detection [9–11, 13, 20, 24, 29–31].

We hypothesized that a single miRNA is not sufficient to predict DLBCL development or to determine a risk profile; instead, a more complete multi-miRNA signature likely needs to be identified to fully detect and potentially target the relevant complex and interacting pathways involved with tumor initiation and development. Leveraging a novel spontaneous DLBCL murine model with Smurf2 deficiency, we were able to identify an age-based key functional circulating miRNA signature that occurs in the blood. This key circulating miRNA signature consists of ten miRNAs (let-7c, let-7b, miR-15a, miR-18a, miR-27a, miR-155, miR-24, miR-130a, miR-10b, and miR-497), which were responsible for DLBCL initiation and was present *prior* to the formation of visible tumor. Quantification of this miRNA signature was done through a cutting edge technology known as droplet digital PCR (ddPCR). Further systems biology analyses revealed that this miRNA signature impacted oncogenic factors related to *JUN* and *MYC* and other important functions associated with cancer cell growth.

## Materials and Methods

### DLBCL spontaneous murine model and collection of blood

We utilized a novel spontaneously producing DLBCL mouse model known as a Smurf2-deficient mice (Smurf2^T/T^, T for the gene-trapping allele) described in detail in previous publications [32, 33]. Age matched C57BL/6 mice were used as wild-type mice and controls. Spleen tissue and bone were harvested using previously described methodology after mice were sacrificed from five Smurf2^T/T^ and wild-type two month old mice [32, 33]. 100 to 200 μl of peripheral blood was harvested from the mice using facial vein blood collection. The blood is allowed to clot for 30 min at room temperature and then centrifuged at 1000×g for 10 min. The serum is removed into a new tube and stored at -80°C until use. These biological replicates were used for each condition: n = 11 young Smurf2^T/T^ mice, n = 12 young wild-type mice, n = 8 old Smurf2^T/T^ mice, and n = 4 old wild-type mice. Young mice are considered to be ~3.5 months old and old mice are ~15 months old. All animal studies were conducted at University of Massachusetts Medical School by Dr. Hong Zhang. At the time of blood collection, all mice were healthy and none of them had visible tumors. For tumors collected for the westerns, mice were sacrificed using standard protocols (isoflurane and cervical dislocation) as referenced in our previous publication [32]. Additionally, spleen and bone marrow were harvested post-mortem from other mice and sacrificed using the same methodology. This study was carried out in strict accordance with the recommendations in the Guide for the Care and Use of Laboratory Animals of the National Institutes of Health. The protocol was approved by the Institutional Animal Care and Use Committee (IACUC) at University of Massachusetts Medical School with protocol number A-1758-15, approved on 4/1/2015. All mice were housed in a fully accredited Animal Care facility and the animal handling and procedures were approved by University of Massachusetts Medical School IACUC.

### miRNA expression profiling on mouse tissue

Expression profiling of miRNA was done on spleen and bone marrow tissue for Smurf2-deficient mice (n = 5) and wild-type (n = 5) at 2 months of age. Microfluidic μParaflo^®^ technology microarray platform (LC Sciences, Houston, TX) was used to obtain the miRNA expression profile. Preprocessing of the miRNA signal was done by standard background subtraction and normalization. The detectable miRNAs were considered by the following two conditions: 1. signal intensity higher than 3×(background standard deviation) and 2. Coefficient of variation (CV) <0.5. CV was calculated by the ratio of standard deviation to signal intensity. When repeating probes are present on an array, the miRNA was listed as detectable only if the signals from at least 50% of the repeating probes are above detection level. Statistically relevant miRNAs were considered with p-values<0.05. Fold changes were determined between the Smurf2-deficient mice and wild-type mice and the average values per group were determined. Network representation of how the miRNAs interact and impact on key transcriptional factors was determined with Ingenuity Pathway Analysis (IPA) software (Ingenuity Systems).

### miRNA isolation

Isolation of circulating miRNAs from serum was done using the QIAgen miRNeasy Mini Kit (QIAgen, CA). Previously published studies revealed that the miRNeasy Mini Kit provides the best yield and highest quality of miRNA from serum [34]. Concentration and quality of miRNAs was quantified by Eppendorf Biophotometer 6131 Spectrophotometer (Eppendorf, Hauppauge, NY).

### cDNA generation and Droplet Digital PCR

From the isolated miRNA, cDNA was first generated using the miRCURY LNA^™^ Universal RT microRNA PCR Universal cDNA Synthesis Kit II (Exiqon, Woburn, MA) using a concentration of 5ng/μl for the miRNA per sample. The miRNA was quantified using the Bio-Rad iCycler Droplet Digital and Real Time PCR system (ddPCR) (Bio-Rad, Hercules, CA). A 1:20 dilution of the generated cDNA was used with the QX200^™^ ddPCR EvaGreen Supermix (Bio-Rad). The following miRCURY LNA^™^ Universal RT microRNA PCR LNA^™^ PCR primer sets (Exiqon) were used with the EvaGreen Supermix: hsa-let-7c-5p, hsa-let-7b-5p, mmu-miR-497-5p, hsa-miR-130a-3p, hsa-miR-18a-5p, hsa-miR-24-3p, hsa-miR-15a-5p, hsa-miR-27a-3p, hsa-miR-10b-5p, and mmu-miR-155-5p. Droplets were generated using the QX200^™^ Automated Droplet Generator (Bio-Rad). The plates were sealed with the PX1^™^ PCR Plate Sealer (Bio-Rad). With the C1000 Touch^™^ Thermal Cycler with 96–Deep Well Reaction Module (Bio-Rad) the following PCR reaction was used for all the primers except for hsa-miR-10b-5p and mmu-miR-155-5p: 1 cycle 95°C for 5 min, 40 cycles of 95°C for 30 sec and 58°C for 1 min (annealing temperature), 1 cycle of 4°C for 5 min, and 1 cycle of 90°C for 5 min. Not all miRNA primers sets for ddPCR will have the same annealing temperature, so optimizing the annealing temperature is required for each primer set. For hsa-miR-10b-5p and mmu-miR-155-5p primer sets the optimal annealing temperatures were 54°C and 52°C. The QX200^™^ Droplet Digital^™^ PCR System (Bio-Rad) quantified the amount of miRNA for each primer set per sample. QuantaSoft software (Bio-Rad) generated the data for each primer set and sample. The same threshold setting was used for all samples per primer set. The concentration (miRNA copies/μl) value generated by QuantaSoft was converted to miRNA copies/ng of serum.

### Predicted miRNA impact on Carcinogenesis, mRNA, and molecular pathways

The impact of this miRNA signature on cancer risk was determined through the literature as to how each miRNA impacts DLBCL (i.e., OncomiR or tumor suppressor). Log_2_ fold-change values were calculated for each miRNA comparing Smurf2^T/T^ to wild-type mice. The overall impact was used to determine the global effect on tumor progression and cancer risk based on the specific regulation of each gene. A *Carcinogenic Risk Score (CRS)* is calculated based on these values to determine if there is a promoted risk for cancer (positive value) or inhibitory risk for cancer (negative value) by summing the log_2_ fold-change values of oncomiRs and subtracting this from the sum of log_2_ fold-change values of the tumor suppressors when comparing either Smurf2^T/T^ to wild-type mice or old to young mice.

CluePedia Cytoscape plugin [35] was used to predict the impact of the miRNA signature on the transcriptome (or mRNA). A threshold of 60 mRNA targets was used when generating the mRNA networks associated with the miRNAs. Additional pathway analysis was done using Ingenuity Pathway Analysis (IPA) software (Ingenuity Systems). The log_2_ fold-change values of the miRNAs comparing Smurf2 deficient to wild-type mice were used to predict through IPA the impact on cancer related functions.

### Western Blot

Total cell lysates were collected using RIPA buffer (50mM Tris-HCl, pH 7.5, 150mM NaCl, 1% Triton X-100, 0.1% SDS, 0.5% deoxycholic acid and 0.02% sodium azide) with freshly added complete protease inhibitors (Roche). Protein lysates (20 mg) were separated by SDS–PAGE Criterion X-gel (Bio-Rad) and transferred to nitrocellulose membranes (GE Osmonics). Immunoblots were visualized using a western lightening chemiluminescence detection kit (Perkin Elmer). The primary antibody was Anti-c-JUN antibody [E254] (Abcam, Cambridge, MA) and anti-GAPDH [FL-335] (Santa Cruz Biotechnology).

## Results

### A common miRNA signature is present in spleen and bone marrow of Smurf2 deficient mice

A novel spontaneous DLBCL murine model was previously established by creating Smurf2-deficient mice (Smurf2^T/T^, T for the gene-trapping allele) with C57BL/6 background [32, 33, 36]. We leveraged this model to study how miRNAs impact tumor formation. Smurf2^T/T^ mice will begin to develop DLBCL starting at 15 months of age [33]. Even though all mice will form DLBCL starting at this age, not all mice manifest DLBCL formation at the same time. The variation in DLBCL formation can range from 15 months to as long as 20 months [33].

**Fig 1:**
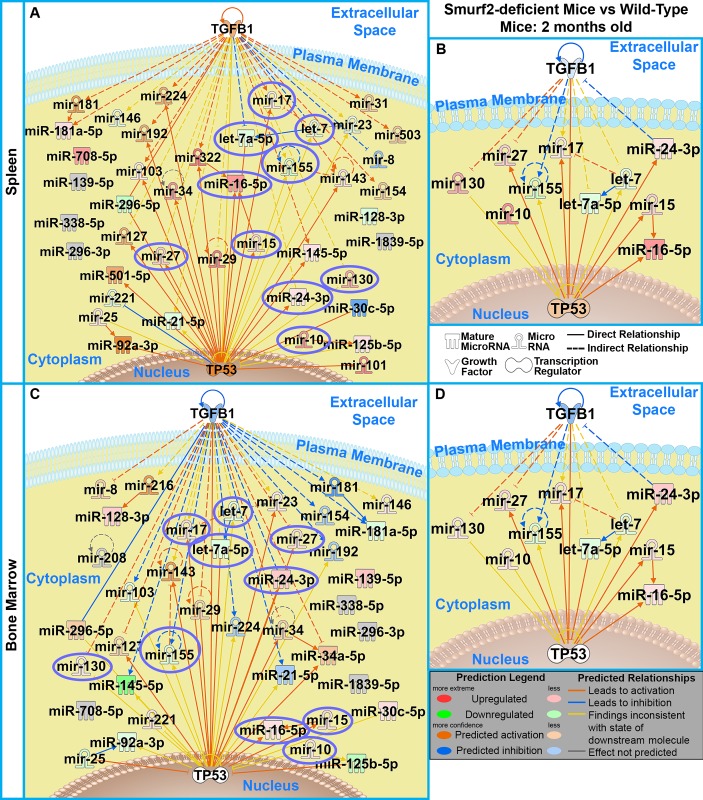
Common miRNA signature in spleen and bone marrow from DLBCL-forming mice. miRNA array was performed on spleen and bone marrow samples from 2 month old mice for both Smurf2 deficient (Smurf2^T/T^) and wild-type (WT) mice. A network generated through IPA of the significantly regulated miRNAs with p-value < 0.05 is shown with the top two most impacted mRNAs (TGFβ1 and p53) for **A)** spleen tissue and **C)** bone marrow comparing Smurf2-deficient mice with wild-type mice. The 10 common miRNAs present in both the **B)** spleen and the **D)** bone marrow are shown as a network with TGFβ1 and p53. The specific miRNA expression values can be found in S1 and S2 Tables.

To investigate the impact of DLBCL initiation on miRNA expression, a profile was performed on both spleen and bone marrow tissue from two month old Smurf2^T/T^ and wild-type mice (S1 and S2 Tables). We determined with Ingenuity Pathway Analysis (IPA) that the significantly regulated miRNAs (*P*<0.05) had the most impact on both *p53* and *TGFβ1* in both the spleen and bone marrow for the Smurf2^T/T^ when compared to the wild-type mice (Fig 1A and 1C). Further analysis led to a surprising discovery of a common miRNA signature being regulated in the same direction between the Smurf2^T/T^ and wild-type mice that is present between both the bone marrow and spleen tissue (Fig 1B and 1D). This miRNA signature consists of 10 miRNAs: miR-130, miR-27, miR-17, miR-10, miR-155, let-7a-5p, let-7, miR-24-3p, miR-15, and miR-16-5p. We previously reported that during tumor progression (and possibly tumor initiation), there is a systemic impact on the host influencing tumor dynamics and molecular factors originating from the host that may be the true “drivers” for carcinogenesis [37, 38]. These data begin to reveal universal drivers (i.e., miRNAs) in the host responsible for DLBCL formation.

### Circulating miRNA signature causing DLBCL formation

The presence of circulating miRNAs in the blood provides a putative mechanism for how a common miRNA signature may impact different areas of the host resulting in DLBCL formation. To examine this theory, we collected peripheral blood from 3.5 months old (“*young”*) and 15 months old (“*old”*) Smurf2^T/T^ and wild-type mice. These ages are roughly equivalent to a 20-year old human for young mice and 50-year old human for the old mice [37]. At the time the serum was prepared none of the old mice had any noticeable tumors. These mice will eventually develop tumors. The expression of 10 common miRNAs predicted above was tested using serum to observe if these miRNAs are circulating through the host. Since miRNAs can have different aliases, the 10 miRNAs (Fig 1) are identified as the following for the rest of this manuscript: let-7 = let-7b, let-7a-5p = let-7c, miR-10 = miR-10b, miR-130 = miR-130a, miR-155 = miR-155, miR-27 = miR27a, miR-24-3p = miR-24, miR-17 = miR-18a, miR-15 = miR-15a, and miR-16-5p = miR-497. Through the use Droplet Digital PCR (ddPCR), we were able to quantify exact counts of circulating miRNA that exists in the serum at high resolution. ddPCR has superior precision over other detection technologies to detect differential expression in miRNAs [39]. Additionally, ddPCR is more resilient to sample quality differences; has a high tolerance to PCR amplification inefficiency; and requires small sample size, which allows for high level of precision being able to capture all known sources of variation [39, 40].

**Fig 2:**
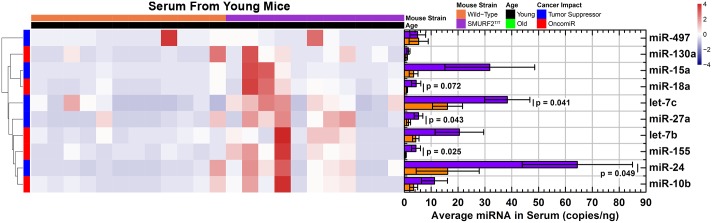
Circulating miRNA signature from the serum of 3-month old mice comparing Smurf2^T/T^ to wild type mice. miRNA was quantified by ddPCR and exact miRNA copies/nanogram of serum was measured. The data is shown as both a heatmap of miRNA copies/nanogram for each mouse and the average counts (bar plots). The error bars in the bar plots represents the standard error. The color gradient for the heatmap represents the miRNA copies/nanogram which is shown as different colors in the hearmap.

From the small amounts of serum, a distinct miRNA signature was captured starting at three months of age, long before actual tumor formation when comparing Smurf2^T/T^ to wild-type mice (Fig 2). All miRNAs, except miR-497, were increased in Smurf2^T/T^ mice compared with wild-type mice (Fig 2). Specifically, let-7c, miR-27a, miR-155, and miR-24 were significantly increased, while the rest of the miRNAs had an increased trend. There was also a large variation between Smurf2^T/T^ mice for most miRNAs, while the wild-type mice exhibit a small variation between individual mice. As stated before, this is due in part that although Smurf2^T/T^ mice start to form DLBCL ~15 months of age, not all mice will form tumors at the same time [33]. In part, since no tumors were present at the time of blood collection for the old mice, we believe that this signature may predict tumor development before it actually forms. This miRNA signature is an indicator of a propensity for future DLBCL growth and can be thought of a risk profile for DLBCL at a young age, which has not previously been described.

**Fig 3:**
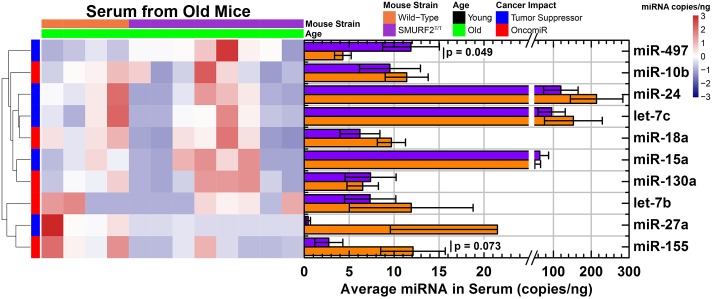
Circulating miRNA signature from the serum of 15-month old mice comparing Smurf2^T/T^ to wild type mice. miRNA was quantified by ddPCR and exact miRNA copies/ng of serum was measured. The data is shown as both a heatmap of miRNA copies/ng for each mouse and the average counts (bar plots). The error bars in the bar plots represents the standard error. The color gradient for the heatmap represents the miRNA copies/nanogram which is shown as different colors in the hearmap.

As the mice approach the age where the DLBCL can start formation (at ~15 months of age), these miRNAs provide a different signature associated with tumor formation. For old mice, miR-497 was significantly increased and miR-155 was significantly decreased for Smurf2^T/T^ compared to wild-type mice (Fig 3). The rest of the circulating miRNAs started to demonstrate a decrease for Smurf2^T/T^ compared to wild-type mice, with exception of miR-130a with minimal change. As with the younger mice, there was a larger variation that occurred for Smurf2^T/T^ mice compared to wild-type mice. The miRNA signature associated with Smurf2^T/T^ mice at this age can be associated with a distinct marker or risk for the presence of DLBCL.

**Fig 4:**
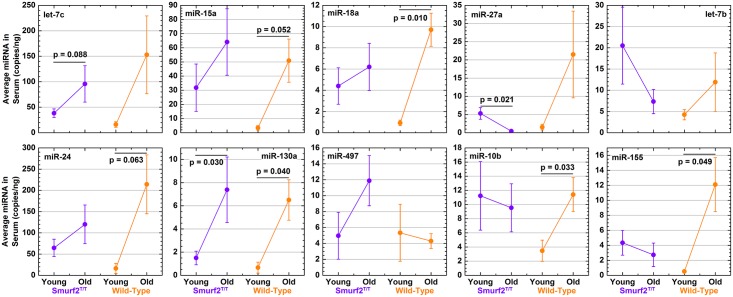
Circulating miRNA signature changing with age in mice susceptible to DLBCL formation compared to wild-type mice. miRNA was quantified by ddPCR and exact miRNA copies/ng of serum was measured. Average miRNA counts are compared between young and old mice for both Smurf2^T/T^ (purple color) and wild-type (orange) groups. The p-values are indicated on the plots and the error bars in the bar plots represents the standard error.

To investigate how the miRNA signature changes with age for mice that will develop DLBCL, comparisons were made between young and old Smurf2^T/T^ mice and compared to the equivalent wild-type mice (Fig 4). Five out of the ten miRNAs (let-7c, miR-15a, miR-18a, miR-24, and miR-130a) showed an increased amount of circulating miRNA with age for both Smurf2^T/T^ and wild-type mice (Fig 4). Interestingly, most of the wild-type mice had a significant increase, while the Smurf2^T/T^ mice only showed the trend. As stated above, the large variance in Smurf2^T/T^ mice was due to the fact that not all mice formed DLBCL at the same time. Thus, these 5 miRNAs were impacted with age regardless of DLBCL formation and the interestingly age-related miRNA signature that they present should be explored further at a future date.

When identifying miRNA changes with age specifically related to Smurf2^T/T^ that differ from the wild-type, miR-27a was the only miRNA which was being suppressed in Smurf2^T/T^ with age, while increasing with age in wild-type age (Fig 4). In the literature, increases in miR-27a have been associated with increased survival for DLBCL patients [12]. The suppression of miR-27a in Smurf2^T/T^ mice with age correlates with the formation of DLBCL and the overall survival of the mice due to the tumor.

### The impact of circulating miRNA on carcinogenic risk

**Fig 5:**
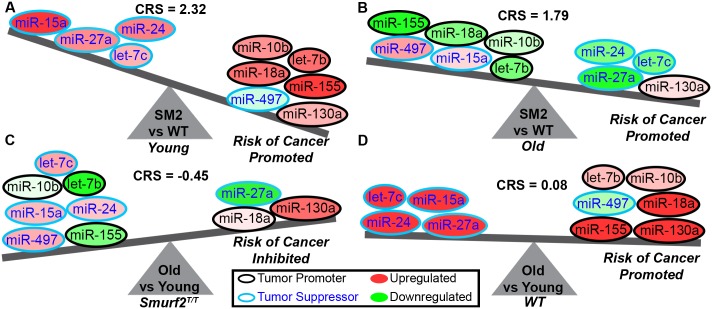
Cancer risk predictions for the circulating miRNA signature. Based on lymphoma literature the miRNAs are classified as tumor promoters (or OncomiRs) and tumor suppressors. Fold-change values are calculated for each miRNA comparing Smurf2^T/T^ (SM2) to wild-type (WT) mice. A Carcinogenic Risk Score (CRS) is calculated based on these values to determine if there is a promoted risk for cancer (positive value) or inhibitory risk for cancer (negative value). **A)** Cancer risk is promoted for young Smurf2^T/T^ mice compared to wild-type. **B)** Cancer risk is promoted for old Smurf2^T/T^ mice compared to wild-type. **C)** Cancer risk is inhibited for Smurf2^T/T^ old mice compared to Smurf2^T/T^ young mice. **D)** Cancer risk is slightly promoted for wild-type old mice compared to wild-type young mice.

A systems biology approach was implemented to determine the overall impact this miRNA signature will have on cancer risk when comparing either Smurf2^T/T^ to wild-type mice or young to old mice. Each of the 10 miRNAs was labeled as a tumor promoter (also referred to as an oncomiR) or a tumor suppressor. The impact of the miRNA was directly associated with DLBCL as opposed to cancer in general, since depending on the cancer type, a miRNA can either be considered as a tumor suppressor or oncomiR (Fig 5). For example, miR-27a for DLBCL has been shown to have overall improved survival for DLBCL patients [12], while in breast cancer, it has been associated as an oncomiR and with poor patient survival [41]. Additionally, let-7b has typically been thought to be a tumor suppressor in most cancers, but it has been shown in DLBCL that let-7b is overexpressed [31, 42] and suggests that it may be considered an oncomiR in this cancer subtype. In addition to miR-27a and let-7b, the following miRNAs from the miRNA signature were considered to be tumor suppressors for DLBCL: miR-15a [29, 32, 43, 44], let-7c [20, 23], miR-24 [12], and miR-497 [9, 45]. The following miRNAs were categorized as oncomiRs for DLBCL: miR-10b [44], miR-155 [9, 19, 29, 44], let-7b [31, 42], miR-18a [1, 14, 41, 44], and miR-130a [44, 46]. A *Carcinogenic Risk Score* (CRS) was subsequently calculated and determines whether the overall risk for cancer will be promoted (if positive) or inhibited (if negative). It is predicted based on this miRNA signature that Smurf2^T/T^ mice for both young (Fig 5A) and old (Fig 5B) age groups have a high positive carcinogenic risk score which indicates the high probability of cancer formation. Interestingly, this miRNA signature was shown to slightly reduce the impact of cancer formation with age (Fig 5C), which indicates that this miRNA signature is more dominant for DLBCL initiation and formation rather than progression. For wild-type mice, there was an overall slight increase risk of cancer based on this miRNA signature when comparing old with young mice (Fig 5D). This indicates that there is an age-related increase risk of cancer.

### The functional impact of circulating miRNA

**Fig 6:**
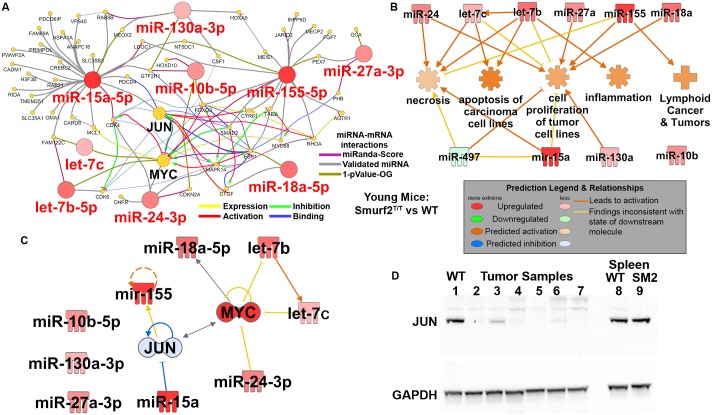
Impact of miRNA signature on transcriptional factors and cancer related function. **A)** For young mice it was determined that the key miRNA signature has impact on *MYC* and *JUN* along with other mRNAs determined using CluePedia Cytoscape plugin. The miRNA-mRNA interactions were determined from three different databases as indicated by the figure legend. **B)** Using Ingenuity Pathway Analysis (IPA) the functional impact of the miRNA signature is shown for young mice comparing Smurf2^T/T^ mice to wild-type (WT) mice. **C)** Using IPA we predicted the impact of the miRNA signature on *JUN* (indicated by blue color being downregulated). *MYC* expression is known to be downregulated in Smurf2^T/T^ mice from previous publications [32]. **D)** Western for *JUN* protein expression on tumor samples from Smurf2^T/T^ mice (lanes 2–7), spleen from wild-type (WT) C57BL/6 mice for the same age the tumor samples (lane 1), and spleen from young wild-type mice (lane 8) and young Smurf2^T/T^ mice (SM2) (lane 9).

Since the existence of this miRNA signature in the host predicted a strong risk of DLBCL, it is important to identify the key functional drivers and targets for these miRNAs. The impact of this miRNA signature on mRNAs was determined by constructing a network of miRNAs interactions with mRNAs (Fig 6A). The miRNA signature network for Smurf2^T/T^ mice versus wild type, revealed *MYC* and *JUN* as key targets (Fig 6A & 6B). Both *MYC* and *JUN* are known to play an important role in DLBCL development and progression [7, 47–49]. The impact on *MYC* and *JUN* from this circulating miRNA signature on the entire host starting at 3 months of age likely act as a trigger for eventual development of DLBCL. We have previously shown that an increase in *MYC* expression is observed in spleens and livers from two month old and also in the fully formed lymphomas from older mice when comparing Smurf2^T/T^ to wild-type C57BL/6 mice [32]. In this publication several methods involving qPCR and westerns were implemented on the different tissue and age groups to demonstrate the increase of *MYC*. We predict using IPA predictive tools that *JUN* signaling should be downregulated due to the impact of the miRNA signature (Fig 6C). Furthermore, we validated the expression of *JUN* in the Smurf2^T/T^ mice compared to the wild-type mice. Western blot revealed a decrease in *JUN* expression in the fully formed lymphomas (from different but identical Smurf2^T/T^ mice which the miRNA signature was measured from) (Fig 6D). Additionally when observing *JUN* expression from the spleen of young mice, we saw no difference in *JUN* signaling when comparing to wild-type (WT) mice (Fig 6D). These *MYC* and *JUN* validations confirm the miRNA predicted impact on these DLBCL related genes.

Finally, using Ingenuity Pathway Analysis (IPA), we were able to predict the impact on significantly regulated biological functions from this miRNA signature (Fig 6B). For young Smurf2^T/T^ mice compared with the wild type, we observed an activation of cancer related functions such as cell proliferation in tumors; inflammation; and lymphoid cancer related functions (Fig 6B). We also noted prominent activation in apoptosis and necrosis. Although cell death related mechanisms were activated at this young age due to the miRNA signature, on balance, the long-term impact of the pro-cancer related functions likely provided sufficient stimulus and drive for the eventual formation of DLBCL.

## Discussion

Understanding of the impact of miRNAs in cancer has greatly evolved since the first links to cancer were observed in early 2000s [50, 51]. More specifically, impact of miRNA on lymphomas was initially underestimated with only a handful of miRNAs, including miR-155 [52, 53] and a miR-16 and miR-15 combination [54], first thought to be the key players in B cell lymphomas. Since then, it has been discovered that a number of other miRNAs, including miR-17 [13, 19], miR-27 [19, 20, 41], miR-24 [19], miR-10 [19, 20], and let-7 [12, 19, 20], play an important role in lymphoma biology. This has led to many possible biomarkers and targets for cancer therapeutics typically using only a single miRNA agent for lymphoma [19, 55].

We employed a systems biology approach to determine a key miRNA signature, and moreover, one that had the most prominent impact on DLBCL development. To explore this, we leveraged a spontaneous DLBCL xenograft model system known as Smurf2^T/T^, whereby 100% of mice develop detectable lymphoma starting at 15 months (and up to 24 months of age). We identified 10 common circulating miRNAs that were expressed in the spleen and bone marrow of mice as early as 2 months of age. For young Smurf2 deficient mice compared with wild type mice, there was a clear predicted risk of cancer promotion due to the miRNA signature detection 15 months *prior to* tumor formation. This miRNA signature was further modulated at older ages associated with the beginning of DLBCL tumor formation. When determining the impact of the miRNA signature on DLBCL biology, it was discovered that these miRNAs had the most prominent impact on genes, *MYC* [48, 49] and *JUN* [7, 47], with global impact on cancer related functions starting at age several months before actual DLBCL formation. We have previously shown that *JUN* is a key driver for DLBCL with aging [8]. In addition, *MYC* is commonly known to be involved DLBCL progression [48]. We have shown in a previous Smurf2^T/T^ related publication that *MYC* expression is elevated in spleen and liver tissue for Smurf2^T/T^ mice started at two months of age and also highly expressed in the eventual formation of the tumor [32]. With the additional data provided in this manuscript showing the decreased expression of *JUN* in the Smurf2^T/T^ mice (Fig 6C & 6D), we have uncovered a possible mechanism for the driving oncogenic factors in DLBCL through the newly identified miRNA signature. We showed herein that there possibly exists a new mechanism in which a DLBCL associated circulating miRNA signature suppresses *JUN* signaling before tumor formation while promoting *MYC* oncogenic factors. Through a simple minimally invasive technique for detecting miRNA signature associated with DLBCL, we were able to predict the oncogenic factors which are known to promote and cause DLBCL. This methodology could conceivably have clinical applications in determining potential treatment strategies before the actual formation for of the tumor.

Currently, early detection of DLBCL is done through non-specific blood markers [56] and/or varied imaging modalities [57]. Current diagnostic methods are only able to detect DLBCL once macroscopic tumor/disease has already developed. This novel miRNA signature could conceivable be used for both determining an early risk profile for DLBCL before formation and it also has potential for developing innovative targeted therapeutic strategies to treat DLBCL.

Recent studies have shown that miRNAs contained in our miRNA signature, such as miR-155, are potentially involved in DLBCL development [58, 59]. Although the extant literature is promising, these studies do not leverage the important consideration of a signature of miRNAs working together concurrently to impact both the development and progression of DLBCL. Furthermore, as shown here, the miRNA signature appeared to mechanistically contribute to DLBCL development even at an early age/point, before frank DLBCL formation. Additionally, it has to be noted that specific miRNAs do not have a universal impact for all cancer types. For example let-7b is generally considered to be a tumor suppressor [60], but when specifically researching let-7b for DLBCL we have found that it actually is associated with tumor promotion and should be considered an oncomiR [31, 42]. Future studies involving specific cancer subtypes should determine the specific impact of the miRNAs on that cancer.

From this early stage, the possibility of cancer prevention can also be implemented by applying a combination of inhibitors for miRNAs (anti-miRs) and miRNA mimetics to mitigate cancer risk. Application of single miRNA related therapy is being studied as a treatment platform [24, 30]. In other cancers using synthetic miRNAs, or mimetics, has demonstrated efficacy with exciting potential for targeted therapy [61, 62]. A single miRNA treatment in murine breast cancer models, such as let-7a or miR-16, resulted in improved survival [43, 63]. While this has shown promising results, it only reduced tumor growth without fully eradicating the tumor. Additionally, the use of anti-miRs to block specific oncomiRs has also shown encouraging results in reduction of tumor growth [64, 65]. To date, therapeutic attempts via modulation of miRNA have been done exclusively with single miRNA targets. It is likely that a combination of miRNA mimetics and anti-miRs together or in combination with other rational targeted therapeutics will be warranted for effective cell kill.

Through our newly discovered miRNA signature, strategic combinations of anti-miR and RNA mimetics may be able to tip the balance from cancer being promoted to inhibited (Figs 5 and 6) by targeting entire pathways and functions regulated by these miRNAs responsible for tumor promotion (Fig 6). Additional work with this miRNA signature should be employed in human studies as potential correlative translational diagnostic assays to test whether it is detectable in DLBCL patients, and moreover, if it is predictive of tumor development or progression. In addition, a future goal might include leveraging such human-specific miRNA signature antagonists in patients with existing DLBCL or with high-risk potential of lymphoma development or progression. Collectively, the overall impact of this work has both short and long-term potential for developing novel cancer biomarkers and potential innovative and highly targeted therapeutic approaches in lymphoma.

## Supporting Information

S1 Table The mean miRNA expression values and p-values from spleen tissue from two month old Smurf2-/- and wild-type mice. (DOCX)

S2 Table The mean miRNA expression values and p-values from bone marrow tissue from two month old Smurf2-/- and wild-type mice. (DOCX)
