# Supplementary material for: A Circulating microRNA Signature Predicts Age-Based Development of Lymphoma
Source: PLoS One. 2017 Jan 20;12(1):e0170521. doi: 10.1371/journal.pone.0170521 (PMC5249061; doi:10.1371/journal.pone.0170521)
Supplement: S1 Table — (DOCX) [file pone.0170521.s001.docx]

**Supplemental Table 1. The mean miRNA expression values and p-values from spleen tissue from two month old Smurf2-/- and wild-type mice.**

| Spleen | p-value | Group 1: Wild-Type  Mean | StDev | Group 2: Knockout  Mean | StDev | Log2 (G2/G1) |
| --- | --- | --- | --- | --- | --- | --- |
| Reporter Name |  |  |  |  |  |  |
| mmu-miR-1937a | 7.00E-09 | 1,956 | 116 | 550 | 37 | -1.83 |
| mmu-miR-709 | 2.15E-08 | 62,981 | 747 | 41,760 | 874 | -0.59 |
| mmu-miR-680 | 3.00E-08 | 1,248 | 78 | 401 | 30 | -1.64 |
| mmu-miR-1195 | 2.25E-07 | 848 | 75 | 238 | 29 | -1.83 |
| mmu-miR-143 | 5.23E-07 | 8,916 | 245 | 11,651 | 247 | 0.39 |
| mmu-miR-1937b | 1.74E-06 | 2,140 | 83 | 657 | 66 | -1.70 |
| mmu-miR-2133 | 2.46E-06 | 1,703 | 113 | 937 | 37 | -0.86 |
| mmu-miR-2132 | 2.51E-06 | 7,864 | 409 | 12,311 | 341 | 0.65 |
| mmu-miR-24 | 3.57E-06 | 8,458 | 185 | 10,076 | 202 | 0.25 |
| mmu-miR-690 | 4.42E-06 | 9,519 | 388 | 13,180 | 547 | 0.47 |
| mmu-miR-483 | 4.77E-06 | 731 | 66 | 311 | 38 | -1.23 |
| mmu-miR-99a | 6.66E-06 | 331 | 21 | 560 | 43 | 0.76 |
| mmu-miR-451 | 1.06E-05 | 27,918 | 315 | 21,848 | 641 | -0.35 |
| mmu-miR-130a | 1.62E-05 | 276 | 22 | 496 | 50 | 0.84 |
| mmu-miR-1892 | 1.63E-05 | 4,230 | 330 | 2,526 | 129 | -0.74 |
| mmu-miR-1187 | 2.38E-05 | 1,628 | 247 | 3,847 | 492 | 1.24 |
| mmu-miR-30d | 2.46E-05 | 4,099 | 258 | 5,957 | 349 | 0.54 |
| mmu-miR-16 | 2.49E-05 | 33,157 | 524 | 30,153 | 445 | -0.14 |
| mmu-let-7d* | 2.76E-05 | 634 | 116 | 206 | 42 | -1.62 |
| mmu-miR-15b | 2.99E-05 | 21,929 | 661 | 18,598 | 443 | -0.24 |
| mmu-miR-27a | 3.84E-05 | 4,539 | 228 | 6,472 | 471 | 0.51 |
| mmu-miR-92b | 4.07E-05 | 6,434 | 181 | 8,193 | 357 | 0.35 |
| mmu-miR-26a | 4.18E-05 | 34,547 | 743 | 29,849 | 890 | -0.21 |
| mmu-miR-2137 | 4.32E-05 | 2,402 | 124 | 1,857 | 70 | -0.37 |
| mmu-miR-2140 | 4.44E-05 | 2,099 | 225 | 1,087 | 134 | -0.95 |
| mmu-miR-486 | 6.02E-05 | 6,181 | 260 | 4,413 | 283 | -0.49 |
| mmu-miR-125b-5p | 7.14E-05 | 9,411 | 396 | 11,574 | 436 | 0.30 |
| mmu-miR-17 | 1.03E-04 | 4,101 | 146 | 4,917 | 183 | 0.26 |
| mmu-miR-155 | 1.15E-04 | 10,470 | 750 | 7,122 | 608 | -0.56 |
| mmu-miR-181a | 1.27E-04 | 3,727 | 166 | 4,675 | 229 | 0.33 |
| mmu-miR-720 | 1.41E-04 | 1,849 | 114 | 1,211 | 116 | -0.61 |
| mmu-let-7c | 1.51E-04 | 29,174 | 1,034 | 24,684 | 888 | -0.24 |
| mmu-miR-2142 | 1.80E-04 | 35,688 | 858 | 31,927 | 786 | -0.16 |
| mmu-miR-484 | 1.84E-04 | 431 | 29 | 594 | 42 | 0.46 |
| mmu-miR-223 | 1.95E-04 | 2,644 | 384 | 4,669 | 526 | 0.82 |
| mmu-miR-107 | 2.04E-04 | 3,314 | 60 | 3,770 | 119 | 0.19 |
| mmu-miR-222 | 2.42E-04 | 1,684 | 88 | 1,272 | 92 | -0.40 |
| mmu-miR-1224 | 2.43E-04 | 16,280 | 426 | 14,127 | 548 | -0.20 |
| mmu-miR-1895 | 2.61E-04 | 2,014 | 232 | 1,183 | 164 | -0.77 |
| mmu-miR-103 | 2.78E-04 | 3,903 | 97 | 4,631 | 209 | 0.25 |
| mmu-let-7a | 2.86E-04 | 33,042 | 993 | 29,233 | 819 | -0.18 |
| mmu-let-7b | 3.59E-04 | 22,361 | 889 | 19,067 | 720 | -0.23 |
| mmu-miR-199a-3p | 3.68E-04 | 2,735 | 155 | 3,812 | 338 | 0.48 |
| mmu-miR-1196 | 4.05E-04 | 3,531 | 255 | 2,489 | 253 | -0.50 |
| mmu-miR-28 | 4.60E-04 | 198 | 29 | 383 | 76 | 0.95 |
| mmu-miR-2146 | 4.60E-04 | 3,063 | 211 | 4,334 | 94 | 0.50 |
| mmu-miR-574-5p | 4.62E-04 | 2,170 | 413 | 3,948 | 515 | 0.86 |
| mmu-miR-21 | 5.44E-04 | 28,195 | 1,030 | 24,218 | 1,062 | -0.22 |
| mmu-miR-19b | 5.58E-04 | 1,258 | 89 | 1,605 | 62 | 0.35 |
| mmu-let-7f | 5.86E-04 | 32,926 | 638 | 29,125 | 1,093 | -0.18 |
| mmu-miR-23b | 6.24E-04 | 22,814 | 568 | 20,737 | 552 | -0.14 |
| mmu-miR-762 | 6.35E-04 | 12,322 | 581 | 14,348 | 497 | 0.22 |
| mmu-miR-26b | 6.73E-04 | 21,753 | 864 | 18,392 | 975 | -0.24 |
| mmu-miR-148a | 7.80E-04 | 1,077 | 162 | 607 | 115 | -0.83 |
| mmu-miR-29a | 9.03E-04 | 17,537 | 471 | 19,235 | 503 | 0.13 |
| mmu-miR-15a | 9.71E-04 | 3,689 | 346 | 5,144 | 515 | 0.48 |
| mmu-miR-23a | 1.15E-03 | 22,304 | 401 | 20,446 | 581 | -0.13 |
| mmu-miR-150 | 1.18E-03 | 39,778 | 1,142 | 35,773 | 1,273 | -0.15 |
| mmu-miR-20a | 1.48E-03 | 5,009 | 260 | 5,904 | 303 | 0.24 |
| mmu-miR-1894-3p | 1.52E-03 | 9,994 | 721 | 7,912 | 609 | -0.34 |
| mmu-miR-146a | 1.56E-03 | 19,863 | 533 | 17,965 | 650 | -0.14 |
| mmu-miR-106b | 1.79E-03 | 2,084 | 70 | 2,365 | 112 | 0.18 |
| mmu-miR-195 | 2.11E-03 | 6,972 | 316 | 8,099 | 430 | 0.22 |
| mmu-miR-128 | 2.16E-03 | 1,215 | 72 | 915 | 103 | -0.41 |
| mmu-miR-29c | 2.30E-03 | 326 | 89 | 786 | 269 | 1.27 |
| mmu-let-7d | 2.60E-03 | 27,627 | 686 | 25,239 | 934 | -0.13 |
| mmu-miR-30e | 2.66E-03 | 650 | 115 | 997 | 97 | 0.62 |
| mmu-miR-290-5p | 3.25E-03 | 2,170 | 148 | 1,062 | 314 | -1.03 |
| mmu-miR-705 | 3.39E-03 | 10,502 | 385 | 9,470 | 365 | -0.15 |
| mmu-miR-2138 | 3.49E-03 | 14,511 | 835 | 12,395 | 702 | -0.23 |
| mmu-miR-99b | 3.78E-03 | 1,990 | 99 | 1,766 | 57 | -0.17 |
| mmu-miR-29b | 4.15E-03 | 271 | 76 | 583 | 188 | 1.10 |
| mmu-miR-423-5p | 4.91E-03 | 3,742 | 208 | 4,608 | 411 | 0.30 |
| mmu-miR-30a | 5.13E-03 | 2,561 | 274 | 3,486 | 459 | 0.45 |
| mmu-miR-425 | 5.25E-03 | 1,635 | 79 | 1,989 | 176 | 0.28 |
| mmu-miR-2134 | 5.66E-03 | 9,239 | 605 | 7,983 | 314 | -0.21 |
| mmu-miR-126-3p | 7.53E-03 | 16,910 | 358 | 17,733 | 348 | 0.07 |
| mmu-miR-151-5p | 7.60E-03 | 3,873 | 137 | 4,175 | 98 | 0.11 |
| mmu-miR-671-5p | 9.05E-03 | 554 | 93 | 380 | 48 | -0.54 |
| mmu-miR-497 | 1.10E-05 | 209 | 16 | 408 | 46 | 0.96 |
| mmu-miR-188-5p | 5.11E-05 | 193 | 31 | 77 | 12 | -1.32 |
| mmu-miR-1897-5p | 1.31E-04 | 138 | 31 | 51 | 11 | -1.43 |
| mmu-miR-1839-3p | 1.45E-04 | 157 | 16 | 40 | 12 | -1.98 |
| mmu-miR-351 | 1.53E-04 | 236 | 20 | 340 | 25 | 0.53 |
| mmu-miR-1966 | 1.66E-04 | 130 | 38 | 39 | 11 | -1.73 |
| mmu-miR-34a | 1.73E-04 | 209 | 23 | 432 | 14 | 1.05 |
| mmu-miR-101b | 1.98E-04 | 95 | 14 | 187 | 31 | 0.98 |
| mmu-miR-199a-5p | 2.84E-04 | 170 | 23 | 301 | 41 | 0.82 |
| mmu-miR-1907 | 2.88E-04 | 161 | 23 | 78 | 16 | -1.05 |
| mmu-miR-466f-3p | 5.95E-04 | 122 | 36 | 38 | 12 | -1.66 |
| mmu-miR-138 | 7.83E-04 | 65 | 11 | 103 | 11 | 0.67 |
| mmu-miR-744 | 1.10E-03 | 162 | 20 | 99 | 16 | -0.71 |
| mmu-miR-340-5p | 1.27E-03 | 139 | 28 | 282 | 31 | 1.02 |
| mmu-miR-328 | 1.56E-03 | 195 | 12 | 315 | 50 | 0.69 |
| mmu-miR-296-5p | 1.75E-03 | 145 | 21 | 94 | 13 | -0.63 |
| mmu-miR-122 | 1.79E-03 | 127 | 18 | 27 | 13 | -2.24 |
| mmu-miR-187 | 2.35E-03 | 229 | 31 | 327 | 18 | 0.51 |
| mmu-miR-10b | 2.35E-03 | 120 | 27 | 245 | 64 | 1.03 |
| mmu-miR-501-3p | 2.53E-03 | 171 | 20 | 238 | 25 | 0.48 |
| mmu-miR-485* | 3.06E-03 | 120 | 18 | 64 | 17 | -0.91 |
| mmu-miR-101a | 4.39E-03 | 43 | 12 | 99 | 35 | 1.20 |
| mmu-miR-327 | 4.87E-03 | 80 | 11 | 40 | 13 | -0.98 |
| mmu-miR-712* | 5.07E-03 | 119 | 14 | 76 | 16 | -0.66 |
| mmu-miR-676 | 5.12E-03 | 187 | 22 | 253 | 30 | 0.44 |
| mmu-miR-467f | 5.62E-03 | 72 | 19 | 30 | 12 | -1.28 |
| mmu-miR-685 | 5.93E-03 | 156 | 18 | 94 | 23 | -0.73 |
| mmu-miR-322 | 6.33E-03 | 94 | 23 | 238 | 106 | 1.33 |
| mmu-miR-466f | 6.47E-03 | 32 | 11 | 77 | 27 | 1.28 |
| mmu-miR-292-5p | 7.59E-03 | 132 | 21 | 85 | 18 | -0.64 |
| mmu-miR-1906 | 9.22E-03 | 71 | 12 | 42 | 11 | -0.77 |
| mmu-miR-197 | 9.53E-03 | 41 | 9 | 24 | 7 | -0.75 |
| mmu-miR-669f | 9.79E-03 | 42 | 19 | 15 | 9 | -1.53 |
| mmu-miR-652 | 1.03E-02 | 337 | 45 | 444 | 31 | 0.40 |
| mmu-miR-2135 | 1.26E-02 | 990 | 220 | 631 | 41 | -0.65 |
| mmu-miR-669n | 1.29E-02 | 53 | 12 | 27 | 12 | -0.99 |
| mmu-miR-145 | 1.47E-02 | 15,542 | 295 | 16,380 | 476 | 0.08 |
| mmu-miR-1944 | 1.49E-02 | 1,170 | 38 | 1,236 | 22 | 0.08 |
| mmu-miR-134 | 1.64E-02 | 68 | 15 | 37 | 12 | -0.87 |
| mmu-miR-466g | 1.77E-02 | 58 | 19 | 28 | 10 | -1.06 |
| mmu-miR-27b | 1.89E-02 | 5,975 | 231 | 6,918 | 624 | 0.21 |
| mmu-miR-674* | 1.94E-02 | 184 | 6 | 264 | 54 | 0.52 |
| mmu-miR-721 | 2.03E-02 | 72 | 26 | 38 | 12 | -0.94 |
| mmu-miR-185 | 2.32E-02 | 1,506 | 78 | 1,374 | 67 | -0.13 |
| mmu-miR-140* | 2.32E-02 | 3,586 | 121 | 3,857 | 173 | 0.11 |
| mmu-miR-342-3p | 2.33E-02 | 9,724 | 252 | 10,161 | 226 | 0.06 |
| mmu-miR-715 | 2.47E-02 | 144 | 26 | 99 | 22 | -0.54 |
| mmu-miR-691 | 2.49E-02 | 82 | 18 | 44 | 20 | -0.89 |
| mmu-miR-100 | 2.59E-02 | 531 | 76 | 707 | 135 | 0.41 |
| mmu-miR-702 | 2.59E-02 | 67 | 11 | 50 | 7 | -0.41 |
| mmu-miR-214 | 2.61E-02 | 3,585 | 230 | 4,017 | 247 | 0.16 |
| mmu-miR-18a | 2.74E-02 | 142 | 6 | 173 | 22 | 0.29 |
| mmu-miR-7a | 2.80E-02 | 201 | 38 | 295 | 71 | 0.55 |
| mmu-miR-145* | 2.86E-02 | 54 | 21 | 101 | 41 | 0.91 |
| mmu-miR-1971 | 3.02E-02 | 77 | 15 | 54 | 15 | -0.52 |
| mmu-miR-674 | 3.16E-02 | 667 | 38 | 745 | 52 | 0.16 |
| mmu-miR-1937c | 3.19E-02 | 103 | 23 | 67 | 22 | -0.61 |
| mmu-miR-703 | 3.20E-02 | 29 | 4 | 19 | 6 | -0.59 |
| mmu-miR-1193 | 3.34E-02 | 28 | 7 | 16 | 6 | -0.85 |
| mmu-miR-350 | 3.48E-02 | 371 | 105 | 544 | 117 | 0.55 |
| mmu-miR-22 | 3.50E-02 | 871 | 68 | 981 | 64 | 0.17 |
| mmu-miR-1 | 3.54E-02 | 6 | 6 | 23 | 15 | 1.92 |
| mmu-miR-872 | 3.74E-02 | 63 | 14 | 92 | 23 | 0.54 |
| mmu-let-7g | 3.76E-02 | 23,773 | 628 | 22,543 | 874 | -0.08 |
| mmu-let-7i | 3.93E-02 | 21,746 | 558 | 20,444 | 957 | -0.09 |
| mmu-miR-1190 | 4.01E-02 | 33 | 6 | 22 | 7 | -0.60 |
| mmu-miR-125a-5p | 4.12E-02 | 5,235 | 514 | 6,115 | 633 | 0.22 |
| mmu-miR-152 | 4.39E-02 | 410 | 59 | 507 | 65 | 0.30 |
| mmu-miR-133b | 4.55E-02 | 23 | 11 | 45 | 11 | 0.96 |
| mmu-miR-199b* | 4.58E-02 | 25 | 5 | 38 | 13 | 0.61 |
| mmu-miR-467e | 4.59E-02 | 7 | 5 | 21 | 10 | 1.61 |
| mmu-miR-93 | 4.83E-02 | 2,659 | 73 | 2,884 | 185 | 0.12 |
| mmu-miR-191 | 4.85E-02 | 16,651 | 286 | 17,165 | 387 | 0.04 |
| mmu-miR-183 | 5.00E-02 | 25 | 6 | 18 | 6 | -0.51 |
| mmu-miR-714 | 5.07E-02 | 188 | 50 | 262 | 46 | 0.48 |
| mmu-miR-2143 | 5.14E-02 | 323 | 27 | 359 | 18 | 0.15 |
| mmu-miR-1940 | 5.18E-02 | 93 | 33 | 132 | 18 | 0.50 |
| mmu-miR-2144 | 5.32E-02 | 95 | 13 | 78 | 11 | -0.29 |
| mmu-miR-98 | 5.42E-02 | 435 | 79 | 543 | 57 | 0.32 |
| mmu-miR-224 | 5.47E-02 | 23 | 6 | 32 | 7 | 0.51 |
| mmu-miR-466j | 5.59E-02 | 31 | 9 | 48 | 16 | 0.63 |
| mmu-miR-20b | 5.60E-02 | 1,373 | 206 | 1,674 | 232 | 0.29 |
| mmu-miR-29b* | 5.80E-02 | 13 | 4 | 22 | 9 | 0.72 |
| mmu-miR-466i | 6.01E-02 | 40 | 12 | 22 | 12 | -0.87 |
| mmu-miR-10a | 6.03E-02 | 1,468 | 156 | 1,241 | 179 | -0.24 |
| mmu-miR-148b | 6.04E-02 | 115 | 31 | 177 | 67 | 0.62 |
| mmu-miR-295* | 6.15E-02 | 28 | 8 | 14 | 8 | -0.97 |
| mmu-let-7e | 6.34E-02 | 7,361 | 1,363 | 9,421 | 1,705 | 0.36 |
| mmu-miR-133a | 6.43E-02 | 40 | 6 | 55 | 15 | 0.47 |
| mmu-miR-194 | 6.43E-02 | 95 | 19 | 118 | 5 | 0.32 |
| mmu-miR-22* | 7.31E-02 | 29 | 13 | 55 | 17 | 0.93 |
| mmu-miR-341 | 7.32E-02 | 71 | 11 | 58 | 10 | -0.31 |
| mmu-miR-467a | 7.34E-02 | 14 | 4 | 25 | 12 | 0.79 |
| mmu-miR-1945 | 7.69E-02 | 29 | 8 | 20 | 5 | -0.55 |
| mmu-miR-491 | 7.72E-02 | 16 | 6 | 27 | 11 | 0.76 |
| mmu-miR-467b | 7.76E-02 | 10 | 10 | 33 | 14 | 1.68 |
| mmu-miR-221 | 8.07E-02 | 907 | 77 | 819 | 55 | -0.15 |
| mmu-miR-1960 | 1.01E-01 | 33 | 5 | 23 | 12 | -0.52 |
| mmu-miR-181b | 1.02E-01 | 391 | 80 | 483 | 66 | 0.30 |
| mmu-miR-200b | 1.03E-01 | 23 | 7 | 31 | 7 | 0.43 |
| mmu-miR-346 | 1.05E-01 | 165 | 40 | 128 | 26 | -0.37 |
| mmu-miR-698 | 1.06E-01 | 37 | 2 | 31 | 7 | -0.29 |
| mmu-miR-92a | 1.10E-01 | 13,310 | 270 | 13,875 | 611 | 0.06 |
| mmu-miR-668 | 1.12E-01 | 59 | 22 | 39 | 15 | -0.61 |
| mmu-miR-700 | 1.13E-01 | 60 | 6 | 74 | 17 | 0.31 |
| mmu-miR-1959 | 1.20E-01 | 110 | 10 | 83 | 34 | -0.40 |
| mmu-miR-151-3p | 1.24E-01 | 651 | 78 | 584 | 11 | -0.16 |
| mmu-miR-467b* | 1.28E-01 | 36 | 13 | 22 | 10 | -0.74 |
| mmu-miR-342-5p | 1.30E-01 | 653 | 41 | 692 | 31 | 0.08 |
| mmu-miR-31 | 1.31E-01 | 55 | 15 | 83 | 35 | 0.59 |
| mmu-miR-712 | 1.36E-01 | 59 | 14 | 48 | 9 | -0.32 |
| mmu-miR-30b | 1.37E-01 | 13,191 | 844 | 12,325 | 760 | -0.10 |
| mmu-miR-30c-1* | 1.39E-01 | 28 | 9 | 41 | 16 | 0.58 |
| mmu-miR-425* | 1.47E-01 | 51 | 8 | 66 | 18 | 0.36 |
| mmu-miR-678 | 1.49E-01 | 53 | 15 | 39 | 14 | -0.46 |
| mmu-miR-383 | 1.52E-01 | 60 | 13 | 47 | 15 | -0.37 |
| mmu-miR-34c* | 1.53E-01 | 56 | 14 | 45 | 8 | -0.32 |
| mmu-miR-210 | 1.55E-01 | 48 | 15 | 62 | 15 | 0.36 |
| mmu-miR-29c* | 1.55E-01 | 24 | 10 | 34 | 12 | 0.52 |
| mmu-miR-296-3p | 1.59E-01 | 29 | 6 | 24 | 3 | -0.27 |
| mmu-miR-1982* | 1.67E-01 | 65 | 19 | 50 | 10 | -0.39 |
| mmu-miR-669c | 1.67E-01 | 184 | 41 | 146 | 38 | -0.33 |
| mmu-miR-411* | 1.67E-01 | 13 | 6 | 21 | 11 | 0.68 |
| mmu-miR-15b* | 1.68E-01 | 68 | 23 | 50 | 11 | -0.46 |
| mmu-miR-1943 | 1.74E-01 | 28 | 13 | 17 | 9 | -0.69 |
| mmu-miR-139-3p | 1.79E-01 | 89 | 22 | 72 | 14 | -0.31 |
| mmu-miR-1941-5p | 1.80E-01 | 105 | 170 | 20 | 4 | -2.39 |
| mmu-miR-186 | 1.84E-01 | 23 | 14 | 33 | 8 | 0.51 |
| mmu-miR-1929 | 1.85E-01 | 29 | 9 | 10 | 9 | -1.46 |
| mmu-miR-291b-5p | 1.87E-01 | 42 | 9 | 31 | 13 | -0.40 |
| mmu-miR-669a | 1.88E-01 | 23 | 13 | 34 | 11 | 0.56 |
| mmu-miR-142-3p | 1.88E-01 | 29 | 8 | 44 | 23 | 0.56 |
| mmu-miR-374 | 1.96E-01 | 1,226 | 375 | 923 | 353 | -0.41 |
| mmu-miR-30e* | 1.97E-01 | 92 | 37 | 129 | 48 | 0.48 |
| mmu-miR-92a* | 2.01E-01 | 30 | 14 | 46 | 26 | 0.62 |
| mmu-miR-713 | 2.01E-01 | 35 | 8 | 26 | 12 | -0.39 |
| mmu-miR-363 | 2.03E-01 | 19 | 9 | 26 | 6 | 0.42 |
| mmu-miR-541 | 2.05E-01 | 10 | 5 | 20 | 13 | 0.94 |
| mmu-miR-466h | 2.05E-01 | 22 | 12 | 32 | 12 | 0.57 |
| mmu-miR-206 | 2.14E-01 | 24 | 6 | 16 | 9 | -0.58 |
| mmu-miR-421 | 2.19E-01 | 138 | 15 | 118 | 29 | -0.22 |
| mmu-miR-532-3p | 2.20E-01 | 32 | 14 | 47 | 19 | 0.57 |
| mmu-miR-1949 | 2.26E-01 | 127 | 2 | 119 | 12 | -0.09 |
| mmu-miR-323-5p | 2.27E-01 | 28 | 16 | 37 | 11 | 0.43 |
| mmu-miR-672 | 2.27E-01 | 44 | 15 | 56 | 17 | 0.37 |
| mmu-miR-30c-2* | 2.29E-01 | 28 | 15 | 37 | 9 | 0.39 |
| mmu-miR-181c | 2.32E-01 | 86 | 64 | 50 | 21 | -0.79 |
| mmu-miR-453 | 2.34E-01 | 8 | 7 | 17 | 15 | 1.07 |
| mmu-miR-105 | 2.36E-01 | 10 | 10 | 19 | 10 | 0.97 |
| mmu-miR-1893 | 2.36E-01 | 31 | 8 | 24 | 5 | -0.35 |
| mmu-miR-532-5p | 2.37E-01 | 601 | 22 | 637 | 56 | 0.08 |
| mmu-miR-681 | 2.44E-01 | 30 | 4 | 22 | 14 | -0.46 |
| mmu-let-7f* | 2.44E-01 | 41 | 10 | 49 | 10 | 0.26 |
| mmu-miR-362-5p | 2.52E-01 | 26 | 9 | 33 | 8 | 0.35 |
| mmu-miR-873 | 2.59E-01 | 13 | 11 | 23 | 11 | 0.79 |
| mmu-miR-15a* | 2.74E-01 | 30 | 16 | 17 | 5 | -0.77 |
| mmu-miR-493 | 2.76E-01 | 22 | 8 | 29 | 13 | 0.44 |
| mmu-miR-339-3p | 2.79E-01 | 23 | 10 | 32 | 16 | 0.46 |
| mmu-miR-509-5p | 2.80E-01 | 9 | 7 | 25 | 27 | 1.38 |
| mmu-miR-764-3p | 2.81E-01 | 13 | 12 | 19 | 10 | 0.54 |
| mmu-miR-1905 | 2.88E-01 | 24 | 5 | 29 | 7 | 0.26 |
| mmu-miR-27b* | 2.89E-01 | 38 | 14 | 49 | 19 | 0.37 |
| mmu-miR-324-3p | 2.89E-01 | 42 | 20 | 30 | 16 | -0.47 |
| mmu-miR-1965 | 2.91E-01 | 28 | 15 | 19 | 10 | -0.58 |
| mmu-miR-200c | 2.91E-01 | 517 | 33 | 495 | 26 | -0.06 |
| mmu-miR-540-5p | 2.95E-01 | 25 | 4 | 20 | 14 | -0.33 |
| mmu-miR-495 | 2.97E-01 | 8 | 8 | 15 | 13 | 0.93 |
| mmu-miR-18b | 3.00E-01 | 11 | 6 | 26 | 27 | 1.25 |
| mmu-miR-1981 | 3.02E-01 | 59 | 14 | 67 | 13 | 0.20 |
| mmu-miR-25 | 3.05E-01 | 11,581 | 626 | 11,123 | 703 | -0.06 |
| mmu-miR-689 | 3.06E-01 | 9,854 | 329 | 9,484 | 674 | -0.06 |
| mmu-miR-142-5p | 3.11E-01 | 693 | 272 | 813 | 149 | 0.23 |
| mmu-miR-149 | 3.13E-01 | 41 | 14 | 50 | 14 | 0.27 |
| mmu-miR-1306 | 3.18E-01 | 110 | 12 | 99 | 23 | -0.15 |
| mmu-miR-203 | 3.20E-01 | 67 | 17 | 76 | 13 | 0.19 |
| mmu-miR-106a | 3.26E-01 | 1,461 | 163 | 1,368 | 89 | -0.09 |
| mmu-miR-542-5p | 3.29E-01 | 11 | 4 | 18 | 12 | 0.73 |
| mmu-miR-1962 | 3.31E-01 | 16 | 9 | 22 | 10 | 0.43 |
| mmu-miR-708 | 3.33E-01 | 28 | 6 | 23 | 9 | -0.27 |
| mmu-miR-1198 | 3.37E-01 | 534 | 38 | 560 | 43 | 0.07 |
| mmu-miR-665 | 3.38E-01 | 40 | 11 | 33 | 15 | -0.27 |
| mmu-miR-378 | 3.40E-01 | 821 | 61 | 782 | 54 | -0.07 |
| mmu-miR-339-5p | 3.45E-01 | 22 | 13 | 33 | 14 | 0.58 |
| mmu-let-7b* | 3.45E-01 | 66 | 21 | 54 | 21 | -0.30 |
| mmu-miR-1930 | 3.46E-01 | 26 | 8 | 22 | 10 | -0.28 |
| mmu-miR-1932 | 3.47E-01 | 15 | 9 | 20 | 9 | 0.45 |
| mmu-miR-883a-3p | 3.59E-01 | 15 | 11 | 11 | 10 | -0.50 |
| mmu-miR-412 | 3.71E-01 | 16 | 12 | 22 | 15 | 0.50 |
| mmu-miR-345-5p | 3.77E-01 | 43 | 12 | 37 | 8 | -0.24 |
| mmu-miR-24-2* | 3.88E-01 | 71 | 23 | 82 | 15 | 0.20 |
| mmu-miR-675-3p | 3.88E-01 | 21 | 4 | 27 | 11 | 0.38 |
| mmu-miR-124* | 3.95E-01 | 12 | 8 | 17 | 13 | 0.49 |
| mmu-miR-543 | 3.98E-01 | 10 | 6 | 16 | 13 | 0.64 |
| mmu-miR-1956 | 4.04E-01 | 48 | 12 | 58 | 22 | 0.27 |
| mmu-miR-219 | 4.04E-01 | 13 | 8 | 17 | 10 | 0.37 |
| mmu-miR-673-3p | 4.08E-01 | 17 | 13 | 21 | 13 | 0.36 |
| mmu-miR-546 | 4.09E-01 | 29 | 4 | 26 | 6 | -0.15 |
| mmu-miR-322* | 4.10E-01 | 129 | 13 | 149 | 43 | 0.21 |
| mmu-miR-1967 | 4.12E-01 | 25 | 11 | 19 | 9 | -0.41 |
| mmu-miR-760 | 4.15E-01 | 27 | 7 | 23 | 11 | -0.23 |
| mmu-miR-763 | 4.21E-01 | 32 | 11 | 466 | 973 | 3.88 |
| mmu-miR-872* | 4.22E-01 | 18 | 9 | 24 | 12 | 0.40 |
| mmu-miR-130b | 4.30E-01 | 710 | 82 | 743 | 47 | 0.07 |
| mmu-miR-449b | 4.34E-01 | 21 | 8 | 18 | 14 | -0.21 |
| mmu-miR-667 | 4.43E-01 | 30 | 10 | 25 | 8 | -0.27 |
| mmu-miR-331-3p | 4.50E-01 | 67 | 15 | 81 | 30 | 0.26 |
| mmu-miR-130b* | 4.53E-01 | 19 | 12 | 13 | 7 | -0.46 |
| mmu-miR-877 | 4.56E-01 | 123 | 33 | 108 | 23 | -0.19 |
| mmu-let-7g* | 4.56E-01 | 24 | 13 | 28 | 11 | 0.25 |
| mmu-miR-1934 | 4.57E-01 | 23 | 12 | 26 | 7 | 0.18 |
| mmu-miR-501-5p | 4.68E-01 | 16 | 6 | 15 | 12 | -0.16 |
| mmu-miR-1983 | 4.71E-01 | 17 | 7 | 22 | 11 | 0.33 |
| mmu-miR-574-3p | 4.79E-01 | 72 | 15 | 77 | 5 | 0.09 |
| mmu-miR-193* | 4.80E-01 | 16 | 4 | 15 | 12 | -0.09 |
| mmu-miR-1947 | 4.83E-01 | 21 | 10 | 17 | 5 | -0.31 |
| mmu-miR-370 | 4.83E-01 | 22 | 12 | 28 | 15 | 0.35 |
| mmu-miR-340-3p | 4.85E-01 | 32 | 14 | 36 | 6 | 0.17 |
| mmu-miR-146b | 4.89E-01 | 1,586 | 444 | 1,404 | 573 | -0.18 |
| mmu-miR-410 | 4.91E-01 | 13 | 6 | 16 | 15 | 0.35 |
| mmu-miR-764-5p | 5.00E-01 | 14 | 12 | 11 | 8 | -0.44 |
| mmu-miR-147 | 5.05E-01 | 11 | 5 | 15 | 11 | 0.44 |
| mmu-miR-30b* | 5.05E-01 | 34 | 7 | 39 | 15 | 0.22 |
| mmu-miR-207 | 5.07E-01 | 29 | 9 | 25 | 14 | -0.18 |
| mmu-miR-338-5p | 5.13E-01 | 24 | 5 | 30 | 16 | 0.33 |
| mmu-miR-28* | 5.17E-01 | 88 | 18 | 100 | 32 | 0.18 |
| mmu-miR-433 | 5.18E-01 | 22 | 5 | 21 | 11 | -0.13 |
| mmu-miR-345-3p | 5.22E-01 | 28 | 13 | 23 | 9 | -0.29 |
| mmu-miR-124 | 5.31E-01 | 18 | 4 | 61 | 94 | 1.72 |
| mmu-miR-361 | 5.34E-01 | 4,393 | 274 | 4,265 | 369 | -0.04 |
| mmu-miR-449c | 5.36E-01 | 15 | 9 | 14 | 14 | -0.05 |
| mmu-miR-409-5p | 5.39E-01 | 13 | 6 | 18 | 11 | 0.47 |
| mmu-miR-139-5p | 5.56E-01 | 3,599 | 267 | 3,710 | 287 | 0.04 |
| mmu-miR-378* | 5.57E-01 | 49 | 12 | 45 | 13 | -0.15 |
| mmu-miR-2182 | 5.67E-01 | 24 | 11 | 26 | 5 | 0.10 |
| mmu-miR-192 | 5.68E-01 | 284 | 63 | 301 | 42 | 0.09 |
| mmu-miR-2141 | 5.76E-01 | 5,780 | 405 | 5,928 | 398 | 0.04 |
| mmu-miR-490 | 5.81E-01 | 14 | 6 | 19 | 11 | 0.46 |
| mmu-miR-669o | 5.83E-01 | 101 | 183 | 24 | 11 | -2.09 |
| mmu-miR-329 | 5.91E-01 | 83 | 22 | 76 | 9 | -0.13 |
| mmu-miR-191* | 5.95E-01 | 42 | 7 | 39 | 16 | -0.09 |
| mmu-miR-718 | 5.98E-01 | 19 | 10 | 22 | 14 | 0.28 |
| mmu-miR-411 | 5.98E-01 | 10 | 5 | 16 | 13 | 0.64 |
| mmu-miR-455 | 6.04E-01 | 802 | 92 | 773 | 72 | -0.05 |
| mmu-miR-692 | 6.08E-01 | 26 | 8 | 31 | 39 | 0.26 |
| mmu-miR-337-5p | 6.13E-01 | 18 | 14 | 23 | 15 | 0.34 |
| mmu-miR-17* | 6.17E-01 | 133 | 16 | 139 | 17 | 0.06 |
| mmu-miR-320 | 6.18E-01 | 4,042 | 122 | 4,123 | 295 | 0.03 |
| mmu-miR-467a* | 6.20E-01 | 37 | 15 | 31 | 14 | -0.24 |
| mmu-miR-1898 | 6.26E-01 | 35 | 8 | 33 | 10 | -0.10 |
| mmu-miR-125a-3p | 6.29E-01 | 54 | 13 | 50 | 18 | -0.11 |
| mmu-miR-298 | 6.29E-01 | 48 | 19 | 51 | 12 | 0.09 |
| mmu-miR-615-5p | 6.39E-01 | 34 | 12 | 29 | 8 | -0.21 |
| mmu-let-7i* | 6.44E-01 | 12 | 7 | 16 | 12 | 0.35 |
| mmu-miR-182 | 6.52E-01 | 33 | 8 | 30 | 11 | -0.12 |
| mmu-miR-150* | 6.54E-01 | 559 | 68 | 575 | 50 | 0.04 |
| mmu-miR-326 | 6.60E-01 | 22 | 7 | 20 | 9 | -0.10 |
| mmu-miR-99b* | 6.61E-01 | 20 | 6 | 22 | 9 | 0.18 |
| mmu-miR-218-2* | 6.70E-01 | 25 | 10 | 21 | 10 | -0.22 |
| mmu-miR-874 | 6.78E-01 | 45 | 17 | 50 | 19 | 0.14 |
| mmu-miR-770-3p | 6.87E-01 | 38 | 19 | 33 | 13 | -0.19 |
| mmu-miR-1199 | 6.92E-01 | 28 | 9 | 26 | 14 | -0.07 |
| mmu-miR-324-5p | 6.94E-01 | 149 | 21 | 144 | 32 | -0.06 |
| mmu-miR-423-3p | 6.94E-01 | 32 | 14 | 39 | 21 | 0.31 |
| mmu-miR-743b-5p | 7.09E-01 | 12 | 8 | 14 | 13 | 0.16 |
| mmu-miR-382 | 7.10E-01 | 23 | 8 | 21 | 9 | -0.10 |
| mmu-miR-470 | 7.14E-01 | 22 | 13 | 19 | 7 | -0.22 |
| mmu-miR-666-5p | 7.19E-01 | 23 | 8 | 24 | 15 | 0.03 |
| mmu-miR-1902 | 7.27E-01 | 19 | 11 | 20 | 7 | 0.08 |
| mmu-miR-30c | 7.34E-01 | 11,859 | 653 | 11,714 | 606 | -0.02 |
| mmu-miR-1896 | 7.38E-01 | 27 | 11 | 24 | 7 | -0.19 |
| mmu-miR-331-5p | 7.40E-01 | 18 | 11 | 20 | 15 | 0.11 |
| mmu-miR-1931 | 7.62E-01 | 40 | 12 | 37 | 11 | -0.10 |
| mmu-miR-127 | 7.62E-01 | 71 | 14 | 72 | 6 | 0.03 |
| mmu-miR-181d | 7.65E-01 | 87 | 27 | 81 | 25 | -0.09 |
| mmu-miR-540-3p | 7.74E-01 | 19 | 12 | 17 | 12 | -0.18 |
| mmu-miR-2145 | 7.79E-01 | 3,638 | 159 | 3,682 | 257 | 0.02 |
| mmu-miR-500 | 7.84E-01 | 27 | 6 | 28 | 14 | 0.02 |
| mmu-miR-1939 | 7.91E-01 | 26 | 14 | 27 | 15 | 0.09 |
| mmu-miR-184 | 7.94E-01 | 37 | 13 | 37 | 11 | 0.03 |
| mmu-miR-381 | 7.95E-01 | 19 | 11 | 39 | 55 | 1.03 |
| mmu-miR-1188 | 7.96E-01 | 37 | 14 | 36 | 15 | -0.04 |
| mmu-miR-483* | 8.09E-01 | 37 | 10 | 39 | 12 | 0.07 |
| mmu-miR-466f-5p | 8.12E-01 | 17 | 11 | 17 | 8 | -0.01 |
| mmu-miR-16* | 8.15E-01 | 22 | 12 | 17 | 7 | -0.38 |
| mmu-miR-770-5p | 8.19E-01 | 23 | 7 | 22 | 10 | -0.06 |
| mmu-miR-805 | 8.32E-01 | 986 | 154 | 962 | 89 | -0.04 |
| mmu-miR-132 | 8.43E-01 | 269 | 28 | 274 | 39 | 0.03 |
| mmu-miR-379 | 8.47E-01 | 38 | 11 | 40 | 13 | 0.05 |
| mmu-miR-675-5p | 8.49E-01 | 20 | 4 | 21 | 15 | 0.10 |
| mmu-miR-1901 | 8.54E-01 | 28 | 12 | 27 | 4 | -0.04 |
| mmu-miR-468 | 8.59E-01 | 18 | 11 | 20 | 12 | 0.13 |
| mmu-miR-196a* | 8.60E-01 | 12 | 7 | 15 | 14 | 0.41 |
| mmu-miR-106b* | 8.70E-01 | 34 | 11 | 34 | 14 | -0.01 |
| mmu-miR-138* | 8.76E-01 | 48 | 41 | 35 | 9 | -0.43 |
| mmu-miR-1935 | 8.77E-01 | 28 | 8 | 28 | 12 | -0.01 |
| mmu-miR-20b* | 8.80E-01 | 19 | 8 | 18 | 13 | -0.03 |
| mmu-miR-135a* | 8.84E-01 | 35 | 9 | 38 | 15 | 0.13 |
| mmu-miR-2139 | 8.91E-01 | 88 | 170 | 10 | 5 | -3.20 |
| mmu-miR-671-3p | 8.95E-01 | 36 | 15 | 32 | 7 | -0.15 |
| mmu-miR-330* | 8.98E-01 | 34 | 10 | 37 | 18 | 0.11 |
| mmu-miR-212 | 9.25E-01 | 21 | 10 | 22 | 16 | 0.12 |
| mmu-miR-7a* | 9.31E-01 | 108 | 26 | 109 | 24 | 0.01 |
| mmu-miR-21* | 9.35E-01 | 25 | 10 | 27 | 17 | 0.08 |
| mmu-miR-27a* | 9.35E-01 | 14 | 5 | 16 | 11 | 0.19 |
| mmu-miR-711 | 9.39E-01 | 21 | 13 | 18 | 7 | -0.20 |
| mmu-miR-290-3p | 9.43E-01 | 50 | 26 | 47 | 14 | -0.10 |
| mmu-miR-129-5p | 9.43E-01 | 26 | 10 | 25 | 5 | -0.08 |
| mmu-miR-1894-5p | 9.54E-01 | 24 | 11 | 21 | 4 | -0.15 |
| mmu-miR-93* | 9.62E-01 | 27 | 8 | 30 | 16 | 0.14 |
| mmu-miR-335-5p | 9.63E-01 | 7 | 5 | 14 | 16 | 1.11 |
| mmu-miR-503 | 9.69E-01 | 15 | 5 | 19 | 13 | 0.31 |
| mmu-miR-30a* | 9.69E-01 | 80 | 17 | 80 | 17 | 0.01 |
| mmu-miR-696 | 9.70E-01 | 21 | 6 | 24 | 16 | 0.22 |
| mmu-miR-409-3p | 9.75E-01 | 38 | 19 | 36 | 17 | -0.07 |
| mmu-miR-1839-5p | 9.78E-01 | 613 | 55 | 619 | 107 | 0.01 |
| mmu-miR-494 | 9.78E-01 | 30 | 15 | 32 | 20 | 0.09 |
| mmu-miR-511 | 9.80E-01 | 7 | 5 | 17 | 14 | 1.16 |
| mmu-miR-193b | 9.88E-01 | 24 | 13 | 23 | 10 | -0.08 |
| mmu-miR-365 | 9.99E-01 | 23 | 5 | 23 | 8 | 0.04 |
