# Supplementary material for: A Circulating microRNA Signature Predicts Age-Based Development of Lymphoma
Source: PLoS One. 2017 Jan 20;12(1):e0170521. doi: 10.1371/journal.pone.0170521 (PMC5249061; doi:10.1371/journal.pone.0170521)
Supplement: S2 Table — (DOCX) [file pone.0170521.s002.docx]

**Supplemental Table 2. The mean miRNA expression values and p-values from bone marrow tissue from two month old Smurf2-/- and wild-type mice.**

| Bone Marrow | p-value | Group 1:  Wild-Type  Mean | StDev | Group 2:  Knockout  Mean | StDev | Log2 (G2/G1) |
| --- | --- | --- | --- | --- | --- | --- |
| Reporter Name |  |  |  |  |  |  |
| mmu-miR-1895 | 2.14E-08 | 1,704 | 278 | 123 | 16 | -3.79 |
| mmu-miR-690 | 4.49E-08 | 5,986 | 187 | 2,455 | 125 | -1.29 |
| mmu-miR-762 | 5.42E-08 | 1,619 | 138 | 404 | 19 | -2.00 |
| mmu-miR-191 | 8.48E-08 | 2,296 | 117 | 4,260 | 147 | 0.89 |
| mmu-miR-1937a | 1.51E-07 | 1,404 | 132 | 449 | 35 | -1.64 |
| mmu-miR-139-5p | 2.25E-07 | 306 | 26 | 956 | 91 | 1.65 |
| mmu-miR-709 | 7.67E-07 | 45,503 | 677 | 53,700 | 505 | 0.24 |
| mmu-miR-451 | 1.42E-06 | 17,136 | 1,084 | 10,278 | 451 | -0.74 |
| mmu-miR-24 | 2.71E-06 | 252 | 28 | 689 | 51 | 1.45 |
| mmu-miR-17 | 2.98E-06 | 412 | 29 | 715 | 41 | 0.80 |
| mmu-miR-2132 | 4.30E-06 | 2,680 | 216 | 1,496 | 91 | -0.84 |
| mmu-miR-1937b | 5.17E-06 | 1,239 | 125 | 449 | 22 | -1.47 |
| mmu-miR-223 | 8.50E-06 | 1,300 | 186 | 8,630 | 350 | 2.73 |
| mmu-miR-30c | 1.80E-05 | 435 | 52 | 840 | 74 | 0.95 |
| mmu-miR-698 | 2.60E-05 | 27 | 11 | 1,346 | 107 | 5.65 |
| mmu-miR-15b | 3.18E-05 | 2,718 | 331 | 6,179 | 290 | 1.18 |
| mmu-miR-92b | 3.82E-05 | 2,277 | 341 | 1,114 | 116 | -1.03 |
| mmu-miR-764-5p | 4.02E-05 | 14 | 6 | 870 | 88 | 5.91 |
| mmu-miR-689 | 6.80E-05 | 163 | 33 | 714 | 27 | 2.13 |
| mmu-miR-361 | 8.43E-05 | 191 | 35 | 439 | 58 | 1.20 |
| mmu-miR-103 | 1.02E-04 | 739 | 76 | 422 | 19 | -0.81 |
| mmu-miR-23a | 1.38E-04 | 1,618 | 158 | 2,401 | 158 | 0.57 |
| mmu-miR-486 | 1.49E-04 | 4,808 | 102 | 4,265 | 125 | -0.17 |
| mmu-miR-26b | 2.55E-04 | 195 | 65 | 1,069 | 60 | 2.46 |
| mmu-miR-26a | 5.01E-04 | 3,719 | 293 | 5,316 | 56 | 0.52 |
| mmu-miR-2146 | 6.40E-04 | 520 | 49 | 271 | 48 | -0.94 |
| mmu-miR-107 | 7.14E-04 | 554 | 59 | 347 | 13 | -0.68 |
| mmu-miR-140* | 8.30E-04 | 868 | 39 | 589 | 66 | -0.56 |
| mmu-miR-574-5p | 8.81E-04 | 550 | 101 | 218 | 63 | -1.34 |
| mmu-miR-92a | 8.94E-04 | 5,581 | 654 | 3,469 | 100 | -0.69 |
| mmu-let-7i | 9.88E-04 | 3,984 | 381 | 2,892 | 122 | -0.46 |
| mmu-miR-705 | 1.00E-03 | 879 | 86 | 631 | 26 | -0.48 |
| mmu-miR-30b | 1.12E-03 | 185 | 46 | 416 | 71 | 1.17 |
| mmu-miR-16 | 1.45E-03 | 6,719 | 341 | 7,904 | 210 | 0.23 |
| mmu-miR-93 | 1.64E-03 | 786 | 69 | 1,008 | 58 | 0.36 |
| mmu-miR-1196 | 2.85E-03 | 434 | 68 | 656 | 54 | 0.60 |
| mmu-miR-20a | 3.01E-03 | 530 | 82 | 865 | 28 | 0.71 |
| mmu-let-7f | 3.24E-03 | 5,224 | 499 | 3,980 | 81 | -0.39 |
| mmu-miR-181a | 6.87E-03 | 796 | 36 | 533 | 89 | -0.58 |
| mmu-miR-320 | 6.93E-03 | 340 | 58 | 491 | 10 | 0.53 |
| mmu-miR-23b | 7.02E-03 | 1,343 | 169 | 1,722 | 114 | 0.36 |
| mmu-let-7a | 7.48E-03 | 5,715 | 792 | 4,227 | 259 | -0.43 |
| mmu-miR-128 | 1.33E-07 | 82 | 6 | 219 | 15 | 1.42 |
| mmu-miR-1944 | 2.83E-06 | 135 | 10 | 273 | 24 | 1.01 |
| mmu-miR-222 | 4.32E-06 | 191 | 18 | 404 | 38 | 1.08 |
| mmu-miR-483 | 1.10E-05 | 248 | 23 | 80 | 13 | -1.63 |
| mmu-miR-1195 | 1.23E-05 | 263 | 16 | 75 | 12 | -1.80 |
| mmu-miR-341 | 1.25E-05 | 302 | 44 | 18 | 7 | -4.08 |
| mmu-miR-685 | 4.17E-05 | 369 | 20 | 25 | 8 | -3.90 |
| mmu-miR-27b | 1.02E-04 | 68 | 7 | 149 | 23 | 1.14 |
| mmu-miR-106b | 1.05E-04 | 167 | 25 | 324 | 37 | 0.96 |
| mmu-miR-125b-5p | 2.33E-04 | 142 | 12 | 53 | 11 | -1.41 |
| mmu-miR-2137 | 2.95E-04 | 422 | 39 | 158 | 35 | -1.42 |
| mmu-miR-2183 | 3.17E-04 | 24 | 9 | 243 | 29 | 3.35 |
| mmu-miR-130b | 5.98E-04 | 87 | 12 | 142 | 19 | 0.70 |
| mmu-miR-1187 | 9.44E-04 | 300 | 55 | 164 | 28 | -0.87 |
| mmu-miR-146a | 1.04E-03 | 166 | 25 | 308 | 65 | 0.89 |
| mmu-miR-712* | 1.04E-03 | 85 | 7 | 128 | 17 | 0.59 |
| mmu-miR-1306 | 1.04E-03 | 86 | 13 | 40 | 11 | -1.12 |
| mmu-miR-145 | 1.15E-03 | 195 | 15 | 33 | 15 | -2.54 |
| mmu-let-7d* | 1.27E-03 | 29 | 8 | 65 | 10 | 1.14 |
| mmu-miR-1949 | 1.33E-03 | 180 | 11 | 46 | 15 | -1.98 |
| mmu-miR-19b | 1.52E-03 | 25 | 10 | 75 | 16 | 1.59 |
| mmu-miR-1839-3p | 1.52E-03 | 49 | 12 | 22 | 7 | -1.19 |
| mmu-miR-30d | 1.70E-03 | 426 | 36 | 311 | 37 | -0.45 |
| mmu-miR-17* | 1.82E-03 | 61 | 14 | 30 | 7 | -1.02 |
| mmu-miR-1894-3p | 2.46E-03 | 291 | 39 | 182 | 37 | -0.68 |
| mmu-miR-27a | 2.54E-03 | 47 | 8 | 73 | 10 | 0.65 |
| mmu-miR-185 | 2.81E-03 | 233 | 20 | 165 | 23 | -0.50 |
| mmu-miR-10a | 3.15E-03 | 41 | 11 | 76 | 12 | 0.89 |
| mmu-miR-342-3p | 3.28E-03 | 78 | 14 | 126 | 16 | 0.70 |
| mmu-miR-221 | 4.57E-03 | 138 | 4 | 215 | 34 | 0.64 |
| mmu-miR-99b | 4.61E-03 | 28 | 8 | 53 | 11 | 0.94 |
| mmu-miR-2145 | 4.82E-03 | 319 | 40 | 235 | 25 | -0.44 |
| mmu-miR-15a | 5.26E-03 | 73 | 16 | 43 | 8 | -0.76 |
| mmu-miR-652 | 6.20E-03 | 52 | 11 | 82 | 14 | 0.66 |
| mmu-miR-805 | 7.02E-03 | 52 | 16 | 95 | 13 | 0.86 |
| mmu-miR-425 | 8.21E-03 | 251 | 23 | 165 | 33 | -0.61 |
| mmu-miR-290-5p | 9.19E-03 | 25 | 11 | 53 | 16 | 1.09 |
| mmu-miR-467f | 1.03E-02 | 57 | 18 | 28 | 10 | -1.04 |
| mmu-miR-1224 | 1.11E-02 | 2,056 | 170 | 2,450 | 193 | 0.25 |
| mmu-miR-681 | 1.25E-02 | 15 | 4 | 27 | 8 | 0.86 |
| mmu-miR-421 | 1.28E-02 | 36 | 16 | 101 | 60 | 1.47 |
| mmu-miR-106a | 1.32E-02 | 72 | 35 | 154 | 20 | 1.09 |
| mmu-miR-1897-5p | 1.38E-02 | 50 | 10 | 27 | 8 | -0.88 |
| mmu-miR-20b | 1.73E-02 | 54 | 17 | 92 | 17 | 0.78 |
| mmu-miR-423-5p | 1.77E-02 | 500 | 54 | 600 | 19 | 0.26 |
| mmu-miR-717 | 1.96E-02 | 9 | 4 | 22 | 9 | 1.40 |
| mmu-miR-540-5p | 2.15E-02 | 20 | 11 | 42 | 7 | 1.08 |
| mmu-miR-718 | 2.22E-02 | 16 | 6 | 29 | 9 | 0.83 |
| mmu-miR-501-3p | 2.42E-02 | 27 | 10 | 47 | 9 | 0.80 |
| mmu-miR-744* | 2.53E-02 | 13 | 8 | 27 | 7 | 1.11 |
| mmu-miR-744 | 2.53E-02 | 30 | 8 | 44 | 2 | 0.56 |
| mmu-miR-702 | 2.61E-02 | 37 | 9 | 58 | 15 | 0.68 |
| mmu-miR-30a | 2.63E-02 | 66 | 15 | 37 | 15 | -0.83 |
| mmu-miR-674 | 2.63E-02 | 106 | 24 | 69 | 18 | -0.62 |
| mmu-miR-674* | 2.74E-02 | 29 | 16 | 66 | 11 | 1.18 |
| mmu-miR-374 | 3.41E-02 | 46 | 17 | 24 | 8 | -0.95 |
| mmu-miR-1894-5p | 3.46E-02 | 40 | 9 | 52 | 5 | 0.39 |
| mmu-miR-133a | 3.53E-02 | 41 | 10 | 27 | 6 | -0.59 |
| mmu-miR-668 | 3.60E-02 | 27 | 15 | 58 | 7 | 1.10 |
| mmu-let-7g | 3.92E-02 | 1,387 | 167 | 1,629 | 100 | 0.23 |
| mmu-miR-485* | 4.01E-02 | 37 | 15 | 65 | 8 | 0.79 |
| mmu-miR-880 | 4.31E-02 | 17 | 8 | 36 | 10 | 1.06 |
| mmu-miR-1193 | 4.50E-02 | 35 | 13 | 55 | 6 | 0.65 |
| mmu-miR-181b | 4.76E-02 | 77 | 23 | 47 | 13 | -0.73 |
| mmu-miR-155 | 4.99E-02 | 174 | 84 | 96 | 13 | -0.85 |
| mmu-miR-2141 | 5.38E-02 | 491 | 77 | 602 | 64 | 0.29 |
| mmu-miR-197 | 5.44E-02 | 51 | 12 | 68 | 5 | 0.42 |
| mmu-let-7d | 5.51E-02 | 3,403 | 416 | 3,922 | 264 | 0.20 |
| mmu-miR-1969 | 5.64E-02 | 25 | 8 | 15 | 6 | -0.72 |
| mmu-miR-181c | 5.84E-02 | 39 | 13 | 25 | 5 | -0.68 |
| mmu-miR-700 | 5.88E-02 | 16 | 12 | 31 | 11 | 0.94 |
| mmu-miR-290-3p | 6.05E-02 | 24 | 9 | 40 | 13 | 0.74 |
| mmu-miR-676 | 6.09E-02 | 26 | 12 | 50 | 22 | 0.94 |
| mmu-miR-872* | 6.22E-02 | 10 | 10 | 124 | 18 | 3.58 |
| mmu-let-7f* | 6.34E-02 | 42 | 8 | 28 | 8 | -0.58 |
| mmu-miR-146b | 6.46E-02 | 43 | 14 | 67 | 19 | 0.66 |
| mmu-miR-1901 | 6.54E-02 | 22 | 10 | 34 | 7 | 0.67 |
| mmu-miR-686 | 7.50E-02 | 12 | 8 | 35 | 10 | 1.58 |
| mmu-miR-1982.1 | 7.52E-02 | 25 | 9 | 36 | 7 | 0.56 |
| mmu-miR-667 | 7.66E-02 | 34 | 17 | 60 | 12 | 0.82 |
| mmu-miR-7a* | 7.70E-02 | 14 | 7 | 32 | 13 | 1.17 |
| mmu-miR-409-3p | 8.37E-02 | 17 | 14 | 143 | 18 | 3.08 |
| mmu-miR-2142 | 8.89E-02 | 19,463 | 458 | 20,345 | 844 | 0.06 |
| mmu-miR-511 | 8.97E-02 | 7 | 13 | 13 | 12 | 0.93 |
| mmu-miR-505 | 9.14E-02 | 11 | 14 | 22 | 5 | 0.99 |
| mmu-miR-188-5p | 9.28E-02 | 25 | 10 | 40 | 15 | 0.70 |
| mmu-miR-692 | 9.70E-02 | 7 | 10 | 30 | 13 | 2.14 |
| mmu-miR-675-3p | 1.05E-01 | 17 | 14 | 36 | 12 | 1.08 |
| mmu-miR-804 | 1.09E-01 | 13 | 6 | 23 | 10 | 0.86 |
| mmu-miR-207 | 1.09E-01 | 19 | 9 | 29 | 9 | 0.61 |
| mmu-miR-106b* | 1.10E-01 | 32 | 10 | 20 | 9 | -0.64 |
| mmu-miR-697 | 1.11E-01 | 5 | 7 | 24 | 13 | 2.35 |
| mmu-miR-1 | 1.13E-01 | 24 | 12 | 6 | 6 | -2.03 |
| mmu-miR-684 | 1.15E-01 | 5 | 7 | 22 | 13 | 1.99 |
| mmu-miR-673-3p | 1.18E-01 | 10 | 8 | 27 | 16 | 1.39 |
| mmu-miR-541 | 1.20E-01 | 10 | 12 | 20 | 11 | 0.97 |
| mmu-miR-488* | 1.22E-01 | 10 | 13 | 21 | 9 | 1.07 |
| mmu-miR-1947 | 1.24E-01 | 564 | 1,212 | 12 | 14 | -5.52 |
| mmu-miR-654-5p | 1.26E-01 | 13 | 11 | 217 | 429 | 4.05 |
| mmu-miR-30e | 1.29E-01 | 28 | 14 | 45 | 10 | 0.69 |
| mmu-let-7e | 1.29E-01 | 128 | 43 | 91 | 9 | -0.50 |
| mmu-miR-615-3p | 1.31E-01 | 13 | 11 | 27 | 10 | 1.04 |
| mmu-miR-466f-3p | 1.37E-01 | 50 | 15 | 29 | 15 | -0.76 |
| mmu-miR-673-5p | 1.38E-01 | 11 | 13 | 27 | 15 | 1.32 |
| mmu-miR-346 | 1.39E-01 | 29 | 14 | 48 | 13 | 0.73 |
| mmu-miR-664 | 1.42E-01 | 25 | 13 | 39 | 14 | 0.64 |
| mmu-miR-455 | 1.43E-01 | 13 | 13 | 26 | 13 | 1.01 |
| mmu-miR-148a* | 1.44E-01 | 23 | 9 | 10 | 11 | -1.29 |
| mmu-miR-149 | 1.44E-01 | 33 | 6 | 28 | 3 | -0.25 |
| mmu-miR-1194 | 1.47E-01 | 25 | 11 | 13 | 7 | -0.90 |
| mmu-miR-299* | 1.48E-01 | 28 | 9 | 38 | 8 | 0.47 |
| mmu-miR-1892 | 1.50E-01 | 91 | 14 | 73 | 19 | -0.32 |
| mmu-miR-704 | 1.51E-01 | 13 | 9 | 25 | 11 | 0.92 |
| mmu-miR-466i | 1.52E-01 | 39 | 16 | 17 | 17 | -1.19 |
| mmu-miR-1943 | 1.54E-01 | 22 | 8 | 13 | 14 | -0.77 |
| mmu-miR-1940 | 1.56E-01 | 28 | 13 | 17 | 11 | -0.77 |
| mmu-miR-654-3p | 1.57E-01 | 11 | 12 | 28 | 10 | 1.33 |
| mmu-miR-199a-3p | 1.58E-01 | 33 | 10 | 48 | 17 | 0.53 |
| mmu-miR-434-3p | 1.60E-01 | 13 | 10 | 25 | 9 | 0.88 |
| mmu-miR-1971 | 1.64E-01 | 24 | 11 | 9 | 8 | -1.40 |
| mmu-miR-181d | 1.67E-01 | 28 | 10 | 35 | 4 | 0.34 |
| mmu-miR-1982.2 | 1.68E-01 | 32 | 13 | 42 | 10 | 0.41 |
| mmu-miR-1935 | 1.71E-01 | 23 | 9 | 14 | 8 | -0.72 |
| mmu-miR-2133 | 1.71E-01 | 60 | 16 | 44 | 15 | -0.47 |
| mmu-miR-665 | 1.72E-01 | 15 | 11 | 32 | 16 | 1.14 |
| mmu-miR-669k | 1.73E-01 | 11 | 10 | 23 | 12 | 1.01 |
| mmu-miR-720 | 1.74E-01 | 254 | 16 | 279 | 30 | 0.13 |
| mmu-miR-615-5p | 1.76E-01 | 16 | 11 | 28 | 11 | 0.77 |
| mmu-miR-670 | 1.82E-01 | 10 | 10 | 28 | 9 | 1.45 |
| mmu-miR-124 | 1.84E-01 | 22 | 11 | 13 | 6 | -0.73 |
| mmu-miR-763 | 1.88E-01 | 25 | 8 | 19 | 12 | -0.46 |
| mmu-miR-669h-3p | 1.89E-01 | 13 | 9 | 26 | 16 | 1.04 |
| mmu-miR-875-5p | 1.94E-01 | 10 | 8 | 26 | 9 | 1.44 |
| mmu-miR-2138 | 1.96E-01 | 385 | 77 | 454 | 76 | 0.24 |
| mmu-miR-329 | 1.97E-01 | 223 | 30 | 194 | 33 | -0.19 |
| mmu-miR-1962 | 2.03E-01 | 25 | 15 | 14 | 12 | -0.86 |
| mmu-miR-1939 | 2.05E-01 | 25 | 9 | 33 | 9 | 0.42 |
| mmu-miR-484 | 2.06E-01 | 94 | 12 | 105 | 15 | 0.17 |
| mmu-miR-22 | 2.07E-01 | 59 | 14 | 44 | 22 | -0.42 |
| mmu-miR-331-3p | 2.08E-01 | 26 | 8 | 18 | 9 | -0.53 |
| mmu-miR-693-5p | 2.12E-01 | 6 | 10 | 24 | 20 | 1.95 |
| mmu-miR-127 | 2.12E-01 | 17 | 11 | 25 | 8 | 0.54 |
| mmu-miR-532-3p | 2.14E-01 | 21 | 8 | 32 | 13 | 0.60 |
| mmu-miR-671-3p | 2.21E-01 | 15 | 13 | 35 | 12 | 1.24 |
| mmu-miR-873 | 2.23E-01 | 10 | 9 | 25 | 13 | 1.27 |
| mmu-miR-1893 | 2.24E-01 | 24 | 10 | 17 | 10 | -0.49 |
| mmu-miR-126-3p | 2.28E-01 | 93 | 11 | 83 | 15 | -0.17 |
| mmu-miR-687 | 2.29E-01 | 9 | 8 | 22 | 10 | 1.23 |
| mmu-miR-669c | 2.29E-01 | 36 | 15 | 25 | 7 | -0.52 |
| mmu-miR-677 | 2.30E-01 | 7 | 8 | 20 | 22 | 1.48 |
| mmu-miR-680 | 2.32E-01 | 44 | 11 | 59 | 20 | 0.44 |
| mmu-miR-1903 | 2.32E-01 | 20 | 11 | 11 | 5 | -0.91 |
| mmu-miR-99a | 2.35E-01 | 17 | 11 | 29 | 14 | 0.80 |
| mmu-miR-30b* | 2.36E-01 | 161 | 318 | 15 | 12 | -3.40 |
| mmu-miR-93* | 2.36E-01 | 14 | 6 | 22 | 11 | 0.69 |
| mmu-miR-205 | 2.37E-01 | 17 | 9 | 27 | 13 | 0.70 |
| mmu-miR-195 | 2.40E-01 | 44 | 10 | 52 | 9 | 0.24 |
| mmu-miR-1970 | 2.42E-01 | 351 | 708 | 23 | 9 | -3.94 |
| mmu-miR-181a-1* | 2.46E-01 | 20 | 11 | 12 | 10 | -0.70 |
| mmu-miR-676* | 2.47E-01 | 10 | 11 | 25 | 18 | 1.33 |
| mmu-miR-1839-5p | 2.47E-01 | 34 | 15 | 42 | 11 | 0.33 |
| mmu-miR-669n | 2.49E-01 | 34 | 18 | 15 | 12 | -1.14 |
| mmu-let-7c | 2.53E-01 | 3,480 | 512 | 3,128 | 366 | -0.15 |
| mmu-miR-193 | 2.54E-01 | 18 | 10 | 13 | 14 | -0.52 |
| mmu-miR-125a-3p | 2.54E-01 | 24 | 11 | 16 | 7 | -0.60 |
| mmu-miR-147 | 2.54E-01 | 19 | 12 | 11 | 13 | -0.76 |
| mmu-miR-16* | 2.54E-01 | 261 | 542 | 11 | 7 | -4.59 |
| mmu-miR-343 | 2.56E-01 | 15 | 11 | 33 | 12 | 1.11 |
| mmu-miR-340-3p | 2.56E-01 | 14 | 11 | 29 | 7 | 1.00 |
| mmu-miR-467a | 2.57E-01 | 15 | 14 | 16 | 11 | 0.09 |
| mmu-miR-18b | 2.58E-01 | 21 | 11 | 11 | 8 | -0.95 |
| mmu-miR-1929 | 2.60E-01 | 22 | 11 | 15 | 11 | -0.58 |
| mmu-miR-669m | 2.64E-01 | 12 | 12 | 19 | 17 | 0.65 |
| mmu-miR-184 | 2.70E-01 | 23 | 14 | 11 | 9 | -1.03 |
| mmu-miR-148b | 2.71E-01 | 28 | 9 | 21 | 17 | -0.38 |
| mmu-miR-696 | 2.85E-01 | 11 | 12 | 23 | 12 | 0.99 |
| mmu-miR-148a | 2.85E-01 | 27 | 11 | 35 | 14 | 0.41 |
| mmu-miR-29a | 2.86E-01 | 417 | 81 | 369 | 25 | -0.17 |
| mmu-miR-143 | 2.87E-01 | 40 | 17 | 29 | 8 | -0.47 |
| mmu-miR-1964 | 2.88E-01 | 23 | 5 | 16 | 15 | -0.53 |
| mmu-miR-1953 | 2.88E-01 | 23 | 10 | 15 | 6 | -0.61 |
| mmu-miR-2134 | 2.91E-01 | 194 | 40 | 226 | 48 | 0.22 |
| mmu-miR-467a* | 2.92E-01 | 20 | 9 | 25 | 6 | 0.32 |
| mmu-miR-323-5p | 2.92E-01 | 27 | 15 | 13 | 11 | -0.99 |
| mmu-miR-2139 | 2.97E-01 | 21 | 12 | 28 | 4 | 0.41 |
| mmu-miR-190 | 2.99E-01 | 18 | 11 | 12 | 13 | -0.50 |
| mmu-let-7c-1* | 3.00E-01 | 21 | 11 | 15 | 15 | -0.45 |
| mmu-miR-15b* | 3.01E-01 | 21 | 14 | 11 | 8 | -0.97 |
| mmu-miR-350 | 3.03E-01 | 18 | 11 | 32 | 8 | 0.78 |
| mmu-miR-186 | 3.06E-01 | 21 | 10 | 14 | 12 | -0.57 |
| mmu-miR-325* | 3.10E-01 | 15 | 15 | 16 | 9 | 0.07 |
| mmu-miR-669i | 3.11E-01 | 12 | 10 | 24 | 15 | 0.98 |
| mmu-miR-1933-5p | 3.17E-01 | 20 | 8 | 15 | 14 | -0.44 |
| mmu-miR-1965 | 3.17E-01 | 23 | 10 | 15 | 10 | -0.61 |
| mmu-miR-2135 | 3.19E-01 | 30 | 14 | 18 | 14 | -0.71 |
| mmu-miR-297a* | 3.19E-01 | 17 | 11 | 26 | 9 | 0.57 |
| mmu-miR-871 | 3.19E-01 | 9 | 8 | 18 | 13 | 0.93 |
| mmu-miR-138* | 3.22E-01 | 187 | 385 | 13 | 7 | -3.90 |
| mmu-miR-468 | 3.26E-01 | 20 | 12 | 14 | 13 | -0.49 |
| mmu-miR-133b | 3.32E-01 | 29 | 7 | 24 | 12 | -0.27 |
| mmu-miR-141* | 3.33E-01 | 21 | 12 | 12 | 9 | -0.73 |
| mmu-miR-2144 | 3.34E-01 | 31 | 10 | 23 | 12 | -0.44 |
| mmu-miR-467e* | 3.35E-01 | 16 | 15 | 21 | 8 | 0.36 |
| mmu-miR-1904 | 3.40E-01 | 22 | 14 | 27 | 6 | 0.30 |
| mmu-miR-21 | 3.41E-01 | 161 | 42 | 135 | 49 | -0.25 |
| mmu-miR-383 | 3.41E-01 | 21 | 12 | 14 | 11 | -0.61 |
| mmu-miR-154* | 3.42E-01 | 19 | 13 | 11 | 6 | -0.80 |
| mmu-miR-33* | 3.46E-01 | 16 | 12 | 23 | 12 | 0.49 |
| mmu-miR-466k | 3.49E-01 | 18 | 11 | 13 | 10 | -0.50 |
| mmu-miR-153 | 3.50E-01 | 19 | 12 | 11 | 9 | -0.76 |
| mmu-miR-21* | 3.50E-01 | 22 | 15 | 11 | 7 | -1.02 |
| mmu-miR-2143 | 3.51E-01 | 31 | 13 | 23 | 16 | -0.46 |
| mmu-miR-19a | 3.53E-01 | 19 | 9 | 14 | 11 | -0.43 |
| mmu-miR-466j | 3.56E-01 | 20 | 10 | 15 | 10 | -0.43 |
| mmu-miR-466l | 3.57E-01 | 14 | 14 | 17 | 13 | 0.25 |
| mmu-miR-376a* | 3.61E-01 | 16 | 13 | 19 | 3 | 0.27 |
| mmu-miR-335-3p | 3.62E-01 | 13 | 11 | 19 | 13 | 0.60 |
| mmu-miR-325 | 3.64E-01 | 21 | 17 | 27 | 12 | 0.35 |
| mmu-miR-19a* | 3.64E-01 | 20 | 7 | 22 | 30 | 0.15 |
| mmu-miR-24-1* | 3.64E-01 | 19 | 11 | 24 | 7 | 0.30 |
| mmu-miR-210 | 3.75E-01 | 26 | 11 | 21 | 17 | -0.32 |
| mmu-miR-345-5p | 3.77E-01 | 40 | 16 | 47 | 7 | 0.22 |
| mmu-miR-150* | 3.80E-01 | 41 | 19 | 30 | 18 | -0.44 |
| mmu-miR-219 | 3.81E-01 | 15 | 10 | 22 | 12 | 0.54 |
| mmu-let-7b | 3.81E-01 | 2,180 | 255 | 2,020 | 302 | -0.11 |
| mmu-miR-701 | 3.82E-01 | 8 | 10 | 16 | 17 | 1.03 |
| mmu-miR-296-5p | 3.85E-01 | 59 | 15 | 50 | 15 | -0.25 |
| mmu-miR-708 | 3.86E-01 | 13 | 7 | 18 | 10 | 0.47 |
| mmu-miR-469 | 3.86E-01 | 14 | 13 | 19 | 15 | 0.40 |
| mmu-miR-669j | 3.87E-01 | 11 | 11 | 25 | 23 | 1.18 |
| mmu-miR-25 | 3.88E-01 | 2,852 | 255 | 2,968 | 142 | 0.06 |
| mmu-miR-1938 | 3.88E-01 | 20 | 10 | 14 | 4 | -0.46 |
| mmu-miR-145* | 3.92E-01 | 20 | 9 | 17 | 18 | -0.22 |
| mmu-miR-300 | 3.94E-01 | 22 | 13 | 16 | 14 | -0.52 |
| mmu-miR-27a* | 3.97E-01 | 13 | 9 | 15 | 18 | 0.18 |
| mmu-miR-760 | 3.99E-01 | 173 | 362 | 19 | 15 | -3.22 |
| mmu-miR-1956 | 4.01E-01 | 20 | 12 | 14 | 11 | -0.46 |
| mmu-miR-666-3p | 4.03E-01 | 9 | 11 | 20 | 17 | 1.11 |
| mmu-miR-467d | 4.04E-01 | 18 | 13 | 20 | 11 | 0.17 |
| mmu-miR-144 | 4.06E-01 | 18 | 13 | 13 | 10 | -0.53 |
| mmu-miR-218-1* | 4.08E-01 | 22 | 12 | 31 | 13 | 0.46 |
| mmu-miR-344 | 4.10E-01 | 17 | 11 | 21 | 8 | 0.30 |
| mmu-miR-186* | 4.12E-01 | 25 | 27 | 15 | 11 | -0.75 |
| mmu-miR-466f-5p | 4.19E-01 | 18 | 14 | 12 | 11 | -0.55 |
| mmu-miR-136* | 4.19E-01 | 19 | 12 | 20 | 7 | 0.11 |
| mmu-miR-142-5p | 4.19E-01 | 24 | 11 | 19 | 12 | -0.36 |
| mmu-miR-758 | 4.20E-01 | 8 | 7 | 13 | 12 | 0.68 |
| mmu-miR-410 | 4.22E-01 | 17 | 17 | 15 | 4 | -0.14 |
| mmu-miR-1190 | 4.25E-01 | 25 | 11 | 20 | 10 | -0.37 |
| mmu-miR-216a | 4.25E-01 | 18 | 12 | 26 | 18 | 0.57 |
| mmu-miR-151-3p | 4.30E-01 | 27 | 13 | 20 | 11 | -0.39 |
| mmu-miR-34a | 4.31E-01 | 26 | 12 | 20 | 11 | -0.40 |
| mmu-miR-1199 | 4.35E-01 | 22 | 8 | 25 | 6 | 0.20 |
| mmu-miR-1898 | 4.35E-01 | 18 | 11 | 15 | 11 | -0.30 |
| mmu-miR-214* | 4.36E-01 | 18 | 9 | 24 | 10 | 0.41 |
| mmu-miR-1936 | 4.37E-01 | 365 | 774 | 19 | 6 | -4.27 |
| mmu-miR-1907 | 4.38E-01 | 29 | 12 | 23 | 12 | -0.33 |
| mmu-miR-199a-5p | 4.39E-01 | 23 | 11 | 28 | 10 | 0.29 |
| mmu-miR-452 | 4.40E-01 | 14 | 13 | 18 | 10 | 0.30 |
| mmu-miR-1-2-as | 4.41E-01 | 14 | 9 | 19 | 13 | 0.48 |
| mmu-miR-770-3p | 4.46E-01 | 17 | 11 | 22 | 13 | 0.39 |
| mmu-miR-1942 | 4.47E-01 | 18 | 9 | 17 | 16 | -0.08 |
| mmu-miR-367 | 4.48E-01 | 22 | 14 | 96 | 180 | 2.16 |
| mmu-miR-1905 | 4.48E-01 | 48 | 10 | 52 | 8 | 0.13 |
| mmu-miR-27b* | 4.48E-01 | 13 | 12 | 14 | 12 | 0.02 |
| mmu-miR-1934 | 4.49E-01 | 25 | 10 | 19 | 7 | -0.37 |
| mmu-miR-26b* | 4.52E-01 | 10 | 12 | 12 | 19 | 0.31 |
| mmu-miR-1966 | 4.52E-01 | 28 | 10 | 36 | 14 | 0.39 |
| mmu-miR-381 | 4.53E-01 | 16 | 13 | 13 | 13 | -0.37 |
| mmu-miR-130b* | 4.54E-01 | 14 | 11 | 20 | 13 | 0.52 |
| mmu-miR-1928 | 4.58E-01 | 17 | 12 | 14 | 9 | -0.27 |
| mmu-miR-2182 | 4.60E-01 | 43 | 13 | 50 | 13 | 0.21 |
| mmu-miR-322* | 4.64E-01 | 23 | 15 | 26 | 11 | 0.21 |
| mmu-miR-669h-5p | 4.64E-01 | 12 | 8 | 25 | 15 | 1.13 |
| mmu-miR-467e | 4.66E-01 | 16 | 17 | 14 | 10 | -0.20 |
| mmu-miR-1188 | 4.68E-01 | 21 | 11 | 57 | 109 | 1.42 |
| mmu-miR-582-5p | 4.72E-01 | 11 | 13 | 17 | 16 | 0.63 |
| mmu-miR-582-3p | 4.78E-01 | 11 | 12 | 17 | 16 | 0.67 |
| mmu-miR-2140 | 4.78E-01 | 85 | 24 | 74 | 17 | -0.20 |
| mmu-miR-490 | 4.82E-01 | 18 | 16 | 19 | 14 | 0.09 |
| mmu-miR-465a-5p | 4.82E-01 | 14 | 13 | 16 | 9 | 0.16 |
| mmu-miR-138 | 4.85E-01 | 19 | 11 | 14 | 9 | -0.37 |
| mmu-miR-191* | 4.88E-01 | 31 | 14 | 24 | 7 | -0.37 |
| mmu-miR-296-3p | 4.93E-01 | 18 | 11 | 9 | 15 | -0.98 |
| mmu-miR-299 | 4.96E-01 | 23 | 13 | 20 | 8 | -0.20 |
| mmu-miR-487b | 4.99E-01 | 16 | 15 | 17 | 9 | 0.13 |
| mmu-miR-1946a | 5.02E-01 | 23 | 10 | 22 | 17 | -0.08 |
| mmu-miR-99b* | 5.05E-01 | 10 | 5 | 34 | 50 | 1.71 |
| mmu-miR-378 | 5.07E-01 | 58 | 12 | 61 | 6 | 0.09 |
| mmu-miR-378* | 5.18E-01 | 20 | 12 | 191 | 383 | 3.24 |
| mmu-miR-338-5p | 5.18E-01 | 21 | 17 | 24 | 13 | 0.22 |
| mmu-miR-34b-5p | 5.20E-01 | 21 | 13 | 15 | 8 | -0.49 |
| mmu-miR-351 | 5.21E-01 | 60 | 18 | 52 | 15 | -0.21 |
| mmu-miR-466g | 5.25E-01 | 23 | 13 | 23 | 16 | 0.00 |
| mmu-let-7b* | 5.26E-01 | 36 | 8 | 32 | 13 | -0.15 |
| mmu-miR-18a | 5.27E-01 | 69 | 39 | 55 | 17 | -0.32 |
| mmu-miR-362-5p | 5.29E-01 | 22 | 13 | 16 | 13 | -0.39 |
| mmu-miR-449c | 5.29E-01 | 17 | 14 | 16 | 16 | -0.05 |
| mmu-miR-141 | 5.37E-01 | 22 | 11 | 18 | 10 | -0.26 |
| mmu-miR-18a* | 5.41E-01 | 21 | 9 | 26 | 11 | 0.29 |
| mmu-miR-345-3p | 5.44E-01 | 25 | 14 | 17 | 10 | -0.52 |
| mmu-miR-465c-5p | 5.47E-01 | 17 | 12 | 12 | 9 | -0.47 |
| mmu-miR-467c | 5.48E-01 | 17 | 13 | 18 | 10 | 0.11 |
| mmu-miR-30c-1* | 5.50E-01 | 22 | 15 | 16 | 10 | -0.46 |
| mmu-miR-1945 | 5.54E-01 | 23 | 10 | 20 | 14 | -0.16 |
| mmu-miR-532-5p | 5.57E-01 | 96 | 13 | 108 | 32 | 0.16 |
| mmu-miR-200a | 5.64E-01 | 18 | 5 | 17 | 13 | -0.08 |
| mmu-miR-666-5p | 5.66E-01 | 18 | 12 | 18 | 11 | 0.06 |
| mmu-miR-669f | 5.68E-01 | 23 | 8 | 29 | 12 | 0.33 |
| mmu-miR-295 | 5.72E-01 | 23 | 14 | 15 | 5 | -0.58 |
| mmu-miR-216b | 5.78E-01 | 28 | 28 | 25 | 9 | -0.17 |
| mmu-miR-34b-3p | 5.83E-01 | 20 | 13 | 20 | 5 | -0.01 |
| mmu-miR-1955 | 5.84E-01 | 72 | 46 | 53 | 20 | -0.44 |
| mmu-miR-375 | 5.88E-01 | 66 | 16 | 61 | 11 | -0.12 |
| mmu-miR-20b* | 5.91E-01 | 18 | 8 | 24 | 15 | 0.45 |
| mmu-miR-703 | 5.94E-01 | 14 | 11 | 17 | 14 | 0.25 |
| mmu-miR-450a-3p | 5.94E-01 | 12 | 13 | 11 | 9 | -0.12 |
| mmu-miR-302d | 5.95E-01 | 51 | 82 | 14 | 10 | -1.84 |
| mmu-miR-134 | 5.97E-01 | 24 | 13 | 26 | 9 | 0.12 |
| mmu-miR-100 | 6.04E-01 | 23 | 11 | 25 | 7 | 0.10 |
| mmu-miR-152 | 6.05E-01 | 24 | 10 | 23 | 13 | -0.10 |
| mmu-miR-30e* | 6.05E-01 | 40 | 56 | 23 | 8 | -0.79 |
| mmu-miR-1941-3p | 6.07E-01 | 25 | 12 | 27 | 9 | 0.10 |
| mmu-miR-678 | 6.08E-01 | 14 | 12 | 23 | 18 | 0.65 |
| mmu-miR-98 | 6.11E-01 | 21 | 15 | 18 | 13 | -0.19 |
| mmu-miR-224 | 6.12E-01 | 22 | 10 | 19 | 11 | -0.20 |
| mmu-miR-342-5p | 6.12E-01 | 38 | 7 | 40 | 6 | 0.08 |
| mmu-miR-483* | 6.20E-01 | 33 | 28 | 32 | 10 | -0.04 |
| mmu-miR-324-5p | 6.23E-01 | 38 | 23 | 28 | 9 | -0.45 |
| mmu-miR-28 | 6.25E-01 | 25 | 12 | 24 | 15 | -0.01 |
| mmu-miR-139-3p | 6.29E-01 | 31 | 13 | 27 | 13 | -0.20 |
| mmu-miR-124* | 6.31E-01 | 18 | 11 | 16 | 10 | -0.22 |
| mmu-miR-1937c | 6.32E-01 | 39 | 12 | 35 | 11 | -0.18 |
| mmu-miR-1198 | 6.32E-01 | 87 | 9 | 91 | 11 | 0.05 |
| mmu-miR-182 | 6.32E-01 | 18 | 12 | 18 | 6 | 0.02 |
| mmu-miR-877 | 6.33E-01 | 20 | 9 | 21 | 6 | 0.10 |
| mmu-miR-449b | 6.38E-01 | 14 | 13 | 16 | 13 | 0.25 |
| mmu-miR-1957 | 6.40E-01 | 23 | 13 | 24 | 14 | 0.09 |
| mmu-miR-291a-5p | 6.50E-01 | 19 | 13 | 19 | 14 | 0.02 |
| mmu-miR-425* | 6.55E-01 | 29 | 18 | 21 | 8 | -0.44 |
| mmu-miR-669g | 6.57E-01 | 12 | 12 | 20 | 16 | 0.67 |
| mmu-miR-140 | 6.58E-01 | 22 | 13 | 22 | 5 | 0.00 |
| mmu-miR-1906 | 6.58E-01 | 25 | 12 | 27 | 10 | 0.12 |
| mmu-miR-129-3p | 6.61E-01 | 58 | 76 | 31 | 11 | -0.92 |
| mmu-miR-151-5p | 6.61E-01 | 64 | 19 | 70 | 21 | 0.13 |
| mmu-miR-669b | 6.65E-01 | 13 | 11 | 16 | 14 | 0.26 |
| mmu-miR-1983 | 6.66E-01 | 17 | 7 | 24 | 16 | 0.45 |
| mmu-miR-220 | 6.66E-01 | 15 | 7 | 24 | 14 | 0.67 |
| mmu-miR-328 | 6.67E-01 | 52 | 11 | 49 | 12 | -0.09 |
| mmu-miR-878-5p | 6.69E-01 | 5 | 4 | 12 | 13 | 1.24 |
| mmu-miR-714 | 6.76E-01 | 23 | 9 | 22 | 11 | -0.09 |
| mmu-miR-327 | 6.79E-01 | 18 | 16 | 11 | 7 | -0.69 |
| mmu-miR-467h | 6.83E-01 | 12 | 11 | 17 | 15 | 0.47 |
| mmu-miR-499 | 6.88E-01 | 10 | 11 | 18 | 14 | 0.86 |
| mmu-miR-215 | 6.96E-01 | 19 | 13 | 18 | 12 | -0.09 |
| mmu-miR-212 | 6.97E-01 | 19 | 12 | 18 | 15 | -0.04 |
| mmu-miR-715 | 6.99E-01 | 43 | 8 | 45 | 7 | 0.05 |
| mmu-miR-326 | 7.06E-01 | 16 | 16 | 22 | 13 | 0.43 |
| mmu-miR-206 | 7.08E-01 | 25 | 9 | 23 | 11 | -0.13 |
| mmu-miR-669d | 7.11E-01 | 14 | 11 | 22 | 13 | 0.72 |
| mmu-miR-423-3p | 7.13E-01 | 24 | 16 | 24 | 13 | 0.02 |
| mmu-let-7g* | 7.14E-01 | 22 | 8 | 21 | 11 | -0.05 |
| mmu-miR-150 | 7.14E-01 | 1,984 | 274 | 2,026 | 153 | 0.03 |
| mmu-miR-411* | 7.16E-01 | 17 | 16 | 14 | 9 | -0.33 |
| mmu-miR-1931 | 7.27E-01 | 24 | 8 | 22 | 9 | -0.10 |
| mmu-miR-872 | 7.27E-01 | 11 | 6 | 18 | 14 | 0.65 |
| mmu-miR-1902 | 7.29E-01 | 21 | 10 | 19 | 8 | -0.15 |
| mmu-miR-382* | 7.35E-01 | 27 | 19 | 26 | 12 | -0.04 |
| mmu-miR-455* | 7.44E-01 | 15 | 13 | 11 | 6 | -0.44 |
| mmu-miR-467b* | 7.45E-01 | 21 | 14 | 21 | 8 | -0.04 |
| mmu-miR-712 | 7.46E-01 | 27 | 11 | 24 | 7 | -0.18 |
| mmu-miR-301a | 7.48E-01 | 27 | 14 | 23 | 8 | -0.23 |
| mmu-miR-675-5p | 7.54E-01 | 9 | 8 | 16 | 16 | 0.86 |
| mmu-miR-130a | 7.57E-01 | 22 | 5 | 21 | 8 | -0.06 |
| mmu-miR-15a* | 7.57E-01 | 19 | 13 | 17 | 5 | -0.18 |
| mmu-miR-20a* | 7.58E-01 | 19 | 9 | 20 | 8 | 0.05 |
| mmu-miR-1954 | 7.58E-01 | 39 | 19 | 38 | 2 | -0.04 |
| mmu-miR-1952 | 7.59E-01 | 29 | 12 | 31 | 12 | 0.12 |
| mmu-miR-295* | 7.59E-01 | 21 | 10 | 18 | 10 | -0.22 |
| mmu-miR-431* | 7.68E-01 | 16 | 14 | 51 | 87 | 1.67 |
| mmu-miR-1967 | 7.70E-01 | 21 | 10 | 24 | 12 | 0.18 |
| mmu-miR-132 | 7.71E-01 | 22 | 11 | 23 | 12 | 0.08 |
| mmu-miR-669l | 7.73E-01 | 12 | 9 | 22 | 14 | 0.81 |
| mmu-miR-218-2* | 7.74E-01 | 19 | 11 | 21 | 14 | 0.14 |
| mmu-miR-590-5p | 7.77E-01 | 25 | 27 | 15 | 9 | -0.75 |
| mmu-miR-1900 | 7.86E-01 | 23 | 9 | 25 | 10 | 0.11 |
| mmu-miR-1899 | 7.87E-01 | 21 | 8 | 32 | 32 | 0.60 |
| mmu-miR-291b-5p | 7.88E-01 | 24 | 7 | 26 | 9 | 0.11 |
| mmu-miR-1948 | 7.96E-01 | 24 | 11 | 23 | 8 | -0.05 |
| mmu-miR-10b | 7.99E-01 | 23 | 13 | 21 | 12 | -0.14 |
| mmu-miR-34c | 8.00E-01 | 19 | 11 | 17 | 11 | -0.19 |
| mmu-miR-96 | 8.04E-01 | 20 | 3 | 27 | 15 | 0.39 |
| mmu-miR-376c | 8.05E-01 | 15 | 14 | 11 | 15 | -0.45 |
| mmu-miR-497 | 8.08E-01 | 11 | 10 | 16 | 15 | 0.52 |
| mmu-miR-470 | 8.11E-01 | 15 | 14 | 13 | 14 | -0.27 |
| mmu-miR-1946b | 8.21E-01 | 39 | 16 | 34 | 9 | -0.18 |
| mmu-miR-193b | 8.31E-01 | 28 | 9 | 29 | 8 | 0.05 |
| mmu-miR-302a* | 8.32E-01 | 15 | 11 | 22 | 16 | 0.51 |
| mmu-miR-200c | 8.43E-01 | 76 | 12 | 79 | 18 | 0.06 |
| mmu-miR-412 | 8.46E-01 | 16 | 15 | 18 | 14 | 0.13 |
| mmu-miR-706 | 8.49E-01 | 23 | 10 | 21 | 8 | -0.11 |
| mmu-miR-30c-2* | 8.55E-01 | 30 | 23 | 16 | 15 | -0.93 |
| mmu-miR-129-5p | 8.56E-01 | 20 | 11 | 20 | 6 | -0.04 |
| mmu-miR-679 | 8.66E-01 | 5 | 6 | 31 | 40 | 2.64 |
| mmu-miR-377 | 8.73E-01 | 13 | 10 | 18 | 19 | 0.46 |
| mmu-miR-29b* | 8.79E-01 | 23 | 19 | 12 | 10 | -0.95 |
| mmu-miR-208a | 8.81E-01 | 18 | 8 | 18 | 9 | -0.02 |
| mmu-miR-192 | 8.84E-01 | 193 | 355 | 58 | 7 | -1.73 |
| mmu-miR-146b* | 8.86E-01 | 19 | 11 | 17 | 7 | -0.15 |
| mmu-miR-574-3p | 8.95E-01 | 124 | 215 | 44 | 10 | -1.48 |
| mmu-miR-363 | 8.96E-01 | 20 | 14 | 23 | 17 | 0.20 |
| mmu-miR-1959 | 8.97E-01 | 22 | 10 | 20 | 8 | -0.14 |
| mmu-miR-382 | 9.04E-01 | 19 | 14 | 16 | 8 | -0.29 |
| mmu-miR-188-3p | 9.05E-01 | 18 | 12 | 23 | 13 | 0.32 |
| mmu-miR-194 | 9.05E-01 | 28 | 14 | 25 | 8 | -0.13 |
| mmu-miR-324-3p | 9.06E-01 | 20 | 17 | 16 | 11 | -0.28 |
| mmu-let-7a* | 9.07E-01 | 18 | 14 | 13 | 7 | -0.43 |
| mmu-miR-214 | 9.13E-01 | 187 | 277 | 96 | 23 | -0.96 |
| mmu-miR-1930 | 9.14E-01 | 21 | 11 | 22 | 13 | 0.08 |
| mmu-miR-493 | 9.18E-01 | 15 | 17 | 18 | 14 | 0.29 |
| mmu-miR-125a-5p | 9.18E-01 | 25 | 14 | 24 | 8 | -0.09 |
| mmu-miR-30a* | 9.22E-01 | 18 | 12 | 19 | 14 | 0.08 |
| mmu-miR-380-3p | 9.24E-01 | 15 | 13 | 16 | 16 | 0.12 |
| mmu-miR-7a | 9.28E-01 | 18 | 12 | 19 | 11 | 0.12 |
| mmu-miR-671-5p | 9.28E-01 | 37 | 15 | 37 | 9 | -0.03 |
| mmu-miR-365 | 9.29E-01 | 25 | 12 | 27 | 14 | 0.16 |
| mmu-miR-431 | 9.31E-01 | 17 | 13 | 17 | 13 | 0.01 |
| mmu-miR-1960 | 9.32E-01 | 21 | 12 | 20 | 12 | -0.10 |
| mmu-miR-491 | 9.34E-01 | 17 | 14 | 22 | 14 | 0.42 |
| mmu-miR-292-5p | 9.42E-01 | 21 | 12 | 22 | 14 | 0.09 |
| mmu-miR-721 | 9.48E-01 | 9 | 7 | 22 | 16 | 1.24 |
| mmu-miR-298 | 9.49E-01 | 29 | 16 | 24 | 4 | -0.25 |
| mmu-miR-28* | 9.59E-01 | 19 | 8 | 21 | 12 | 0.16 |
| mmu-miR-2136 | 9.61E-01 | 23 | 14 | 22 | 13 | -0.09 |
| mmu-miR-370 | 9.64E-01 | 67 | 108 | 24 | 6 | -1.50 |
| mmu-miR-719 | 9.68E-01 | 14 | 7 | 18 | 14 | 0.39 |
| mmu-miR-494 | 9.79E-01 | 16 | 15 | 10 | 7 | -0.66 |
| mmu-miR-294 | 9.93E-01 | 22 | 17 | 16 | 11 | -0.45 |
| mmu-miR-297a | 9.96E-01 | 20 | 11 | 20 | 14 | -0.01 |
| mmu-miR-380-5p | 9.97E-01 | 18 | 16 | 15 | 17 | -0.22 |
| mmu-miR-338-3p | 9.97E-01 | 28 | 26 | 11 | 12 | -1.35 |
